# Reactive Oxygen Species and Antioxidant Defense in Plants under Abiotic Stress: Revisiting the Crucial Role of a Universal Defense Regulator

**DOI:** 10.3390/antiox9080681

**Published:** 2020-07-29

**Authors:** Mirza Hasanuzzaman, M.H.M. Borhannuddin Bhuyan, Faisal Zulfiqar, Ali Raza, Sayed Mohammad Mohsin, Jubayer Al Mahmud, Masayuki Fujita, Vasileios Fotopoulos

**Affiliations:** 1 Department of Agronomy, Faculty of Agriculture, Sher-e-Bangla Agricultural University, Sher-e-Bangla Nagar, Dhaka 1207, Bangladesh; 2 Citrus Research Station, Bangladesh Agricultural Research Institute, Jaintapur, Sylhet 3156, Bangladesh; mhmb_bhuyan@bari.gov.bd; 3 Institute of Horticultural Sciences, Faculty of Agriculture, University of Agriculture Faisalabad, Faisalabad 38000, Pakistan; ch.faisal.zulfiqar@gmail.com; 4 Key Lab of Biology and Genetic Improvement of Oil Crops, Oil Crops Research Institute, Chinese Academy of Agricultural Sciences (CAAS), Wuhan 430062, China; alirazamughal143@gmail.com; 5 Laboratory of Plant Stress Response, Faculty of Agriculture, Kagawa University, Miki-cho, Kita-Gun, Kagawa 761-0795, Japan; mohsin@sau.edu.bd (S.M.M.); fujita@ag.kagawa-u.ac.jp (M.F.); 6 Department of Plant Pathology, Faculty of Agriculture, Sher-e-Bangla Agricultural University, Sher-e-Bangla Nagar, Dhaka 1207, Bangladesh; 7 Department of Agroforestry and Environmental Science, Faculty of Agriculture, Sher-e-Bangla Agricultural University, Sher-e-Bangla Nagar, Dhaka 1207, Bangladesh; jamahmud_bd@yahoo.com; 8 Department of Agricultural Sciences, Biotechnology & Food Science, Cyprus University of Technology, P.O. Box 50329, Lemesos 3603, Cyprus

**Keywords:** abiotic stress, antioxidant systems, ascorbate-glutathione pathway, cross tolerance, H_2_O_2_, oxidative stress, plant stress tolerance, reactive nitrogen species, reactive oxygen species, stress signaling

## Abstract

Global climate change and associated adverse abiotic stress conditions, such as drought, salinity, heavy metals, waterlogging, extreme temperatures, oxygen deprivation, etc., greatly influence plant growth and development, ultimately affecting crop yield and quality, as well as agricultural sustainability in general. Plant cells produce oxygen radicals and their derivatives, so-called reactive oxygen species (ROS), during various processes associated with abiotic stress. Moreover, the generation of ROS is a fundamental process in higher plants and employs to transmit cellular signaling information in response to the changing environmental conditions. One of the most crucial consequences of abiotic stress is the disturbance of the equilibrium between the generation of ROS and antioxidant defense systems triggering the excessive accumulation of ROS and inducing oxidative stress in plants. Notably, the equilibrium between the detoxification and generation of ROS is maintained by both enzymatic and nonenzymatic antioxidant defense systems under harsh environmental stresses. Although this field of research has attracted massive interest, it largely remains unexplored, and our understanding of ROS signaling remains poorly understood. In this review, we have documented the recent advancement illustrating the harmful effects of ROS, antioxidant defense system involved in ROS detoxification under different abiotic stresses, and molecular cross-talk with other important signal molecules such as reactive nitrogen, sulfur, and carbonyl species. In addition, state-of-the-art molecular approaches of ROS-mediated improvement in plant antioxidant defense during the acclimation process against abiotic stresses have also been discussed.

## 1. Introduction

Environmental stresses, including salinity, drought, extreme temperature, toxic metals/metalloids, flooding/waterlogging (WL), etc. are now prevalent due to drastic and harsh climate change [1,2]. The aggravation of such diverse abiotic stresses has become a major threat to sustainable crop production. Alongside, numerous detrimental effects lead to oxidative stress through the overaccumulation of reactive oxygen species (ROS) including free radicals (superoxide anion, O_2_^•−^; hydroperoxyl radical, HO_2_^•^; alkoxy radical, RO^•^; and hydroxyl radical, ^•^OH) and nonradical molecules (hydrogen peroxide, H_2_O_2_ and singlet oxygen, ^1^O_2_) [3,4]. High-energy initiation or electron transfer reactions lead to atmospheric oxygen (O_2_) to the abovementioned partially reduced or activated forms of molecular oxygen [5]. The primary cellular ROS generation sites are chloroplasts, mitochondria, peroxisomes, apoplast, and plasma membranes [6]. Although ROS are formed in the plant as part of normal cellular metabolism, overaccumulation due to stress severely damages necessary cellular ingredients including carbohydrates, proteins, lipids, DNA, etc. because of their highly reactive nature [7].

Plants primarily deal with oxidative stress via an endogenous defensive mechanism consisting of different enzymatic (superoxide dismutase, SOD; catalase, CAT; ascorbate peroxidase, APX; glutathione reductase, GR; monodehydroascorbate reductase, MDHAR; dehydroascorbate reductase, DHAR; glutathione peroxidase, GPX; guaiacol peroxidase, GOPX; glutathione *S*-transferase, GST; Ferritin; nicotinamide adenine dinucleotide phosphate (NADPH) oxidase-like alternative oxidase, AOX; peroxiredoxins, PRXs; thioredoxins, TRXs; glutaredoxin, GRX; etc.) and nonenzymatic (ascorbic acid, AsA; glutathione, GSH; phenolic acids; alkaloids; flavonoids; carotenoids; α-tocopherol; nonprotein amino acids; etc.) antioxidants [8,9,10]. In plant cells, the antioxidant defense system and ROS accumulation uphold a steady-state balance [9]. Maintaining an optimum ROS level in the cell enables proper redox biology reactions and the regulation of numerous processes essential for plants such as growth and development [11]. This intermediate level is maintained by the balance between ROS production and ROS scavenging [4]. However, during stress conditions, overgeneration of ROS demolishes the equilibrium and causes cellular damage, leading to programmed cell death (PCD) as well as decreasing plant productivity [7].

Besides their damaging activity, ROS are well known as secondary messengers or signaling molecules and transport the signal to the nucleus through redox reactions using mitogen-activated protein kinase (MAPK) pathway in a variety of cellular mechanisms to increase tolerance against diverse abiotic stresses [6]. Reactive oxygen species participate as major molecules in the acclimation process of plants under environmental stimuli. They principally act as signal transduction molecules, which control diverse pathways throughout the acclimation of the plant under stress conditions [5,12]. Several studies showed that ROS are essential for the success of numerous fundamental natural processes, including cellular proliferation and differentiation [11]. In addition, H_2_O_2_ is a critical component of stress response regulation in crop plants such as rice [13], wheat [14], maize [15], mung bean [16], soybean [17], cucumber [18], sour orange [19], strawberry [20], basil [21], and rapeseed [22]. Moreover, it is established that in addition to ROS, reactive nitrogen species (RNS), reactive sulfur species (RSS), and reactive carbonyl species (RCS) also play a key signaling role and are all involved in a cross-talk in plant abiotic stress tolerance [23]. Therefore, ROS play a crucial, dual role in plant biology, representing a fascinating area of research for plant biologists.

In this review, we summarize the recent progress of harmful effects of ROS, antioxidant defense system involved in ROS detoxification under different abiotic stresses, and also the cross-talk of RNS, RSS, and RCS with ROS. We also focus on progress in molecular approaches of ROS-mediated improvement in plant antioxidant defense during the acclimation process against abiotic stress.

## 2. Chemistry of Reactive Oxygen Species

**Figure 1:**
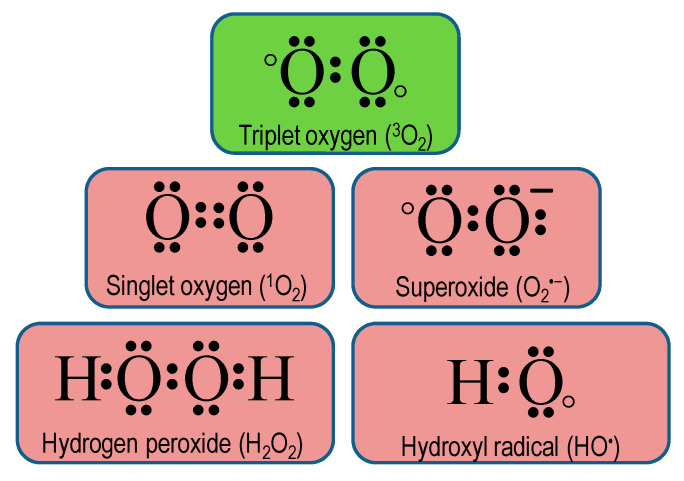
Lewis dot structure of triplet oxygen and reactive oxygen species.

Atmospheric O_2_ is a free molecule that exists in the ground state (triplet oxygen, ^3^O_2_) having two unpaired parallel spin electrons with the same spin numbers, which drop off its reactivity. However, additional energy from some biochemical reactions, electron transport chains (ETC), ultraviolet-B, and ionizing irradiations assist ^3^O_2_ to get rid of the spin restriction and thus becoming ROS (Figure 1) [24].

In plant cells, ROS can be formed in many compartments including chloroplasts, mitochondria, peroxisomes, and plasma membrane [25]. In the chloroplast, the chlorophyll (chl) pigments absorb light quanta and become excited to their triplet state. If this triplet chl is not quenched efficiently, a charge recombination occurs leading ^3^O_2_ to excited ^1^O_2_ [25]. Although its lifetime is very short (3.1–3.9 μs) and diffusion distance is low (190 nm), ^1^O_2_ diffuses outside the chloroplast to reach the cell wall, targets plasma membrane, tonoplast, or even cytosolic signaling cascades [26]. Furthermore, ^3^O_2_ can receive electrons from ETC or nicotinamide adenine dinucleotide phosphate (NADPH) oxidase activity producing O_2_^•−^, which has a half-life of 1–1000 μs [4]. In addition, O_2_^•−^ reacts with H^+^ producing HO_2_^•−^, which is far more reactive, stable, and permeable through biological membranes. Similarly, H_2_O_2_ can be produced through the dismutation of O_2_^•−^/HO_2_^•−^ by SOD isoforms, NADPH oxidases, and heme-containing class III peroxidases (POX) activity [27,28]. Chemically, H_2_O_2_ acts as a weak acid that is highly diffusible and stable, having a lifetime of <1 s, and could cross the plasma membrane via aquaporins [29]. Another important ROS—^•^OH, can be produced by the Fenton reaction, hydroperoxides activity during sunlight, and inner-sphere electron transfer. Moreover, specific proteins, such as heme oxygenases, cytochrome P450s, superoxide reductases, and some photosystem II (PSII) proteins, also generate ^•^OH [30]. The computed half-life of ^•^HO is approximately 1 ns and has a short diffusibility (<1 nm).

Some reactions of production and conversions of ROS in the biological system:

O_2_ + e^−^ → O_2_^•−^ (1)

O_2_^•−^ + H^+^ ⇄ HO_2_^•−^ (2)

H_2_O_2_ + HO^•^ ⇄ HO_2_^•−^ + H_2_O (3)

^•^OH + ^•^OH ⇄ O^•^ + H_2_O (4)

O_2_^•−^ + 2H^+^ + e^−^ → H_2_O_2_ (5)

O_2_^•−^ + HO_2_^•−^ + H_2_O → H_2_O_2_ + O_2_ + ^•^OH (6)

HOOH → HO^•^ + ^•^OH (7)

ROOH → RH^•^ + ^•^OH (8)

Fe^3+^/Cu^2+^/Mn^3+^ + H_2_O_2_ → Fe^2+^/Cu^+^/Mn^2+^ OH + ^•^OH (9)

Fe^2+^/Cu^+^/Mn^2+^ + H_2_O_2_ → Fe^3+^/Cu^2+^/Mn^3+^ + HO_2_^•−^ + H^+^ (10)

**Figure 2:**
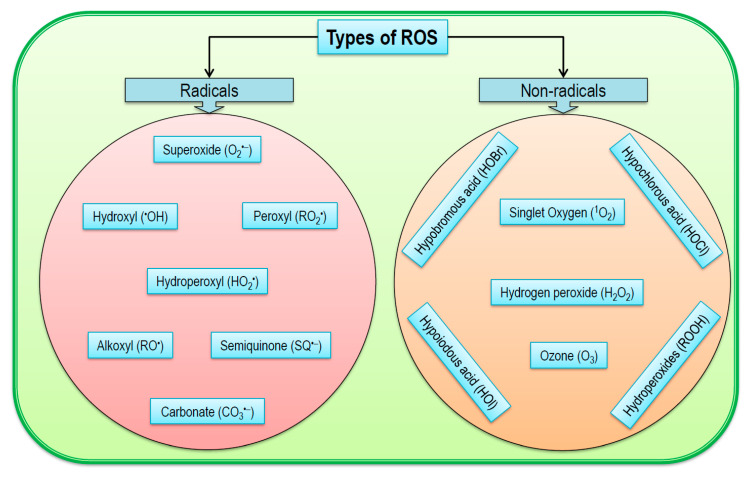
Types of reactive oxygen species in plants.

Cellular ROS are comprised of both free radical and nonradicals (Figure 2). Among the free radicals, O_2_^•−^, ^•^OH, RO^•^, and peroxyl radical (ROO^•^) and nonradicals, H_2_O_2_, ^1^O_2_, and ozone (O_3_) are common [31]. However, some other nonradical ROS are also found in plants such as hypochlorous acid (HOCl), hydroperoxides (ROOH), and excited carbonyls (RO*) [32]. Moreover, reactive oxygen intermediates (ROI) are also classified as reactive oxygen molecules formed by incomplete reduction of O_2_; therefore, ROS include all types of ROI as well as O_3_ and ^1^O_2_. In addition, some acids like hypobromous acid (HOBr), hypoiodous acid (HOI), and HOCl and radicals like carbonate radical (CO_3_^•−^) and semiquinone (SQ^•−^) are also incorporated into ROS [33,34,35].

Among ROS, O_2_^•−^ predominantly acts as a reducing agent forming strong oxidants. Moreover, O_2_^•−^ reacts with nitric oxide (NO) producing RNSs, RSSs, and RCSs. These compounds also promote oxidative stress, and are involved in “shaping” the intra- and extracellular redox signal [36,37].

## 3. Localization and Processes of the Generation of ROS in Plant Cells

**Figure 3:**
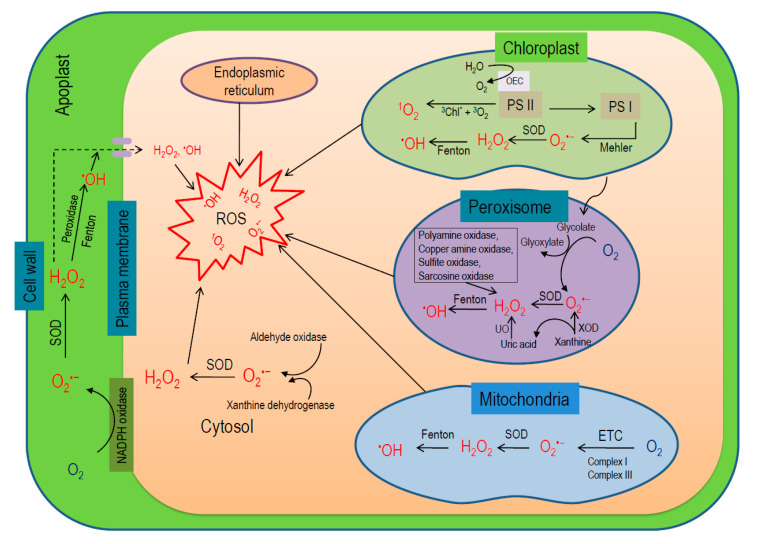
Localization and processes for the generation of ROS in plant cells (ROS, reactive oxygen species; H_2_O_2_, hydrogen peroxide; O_2_^•−^, superoxide anion; ^1^O_2_, singlet oxygen; ^•^OH, hydroxyl radical; SOD, superoxide dismutase; UO, urate oxidase; XOD, xanthine oxidase; ETC, electron transport chain; PS I, photosystem I; PS II, photosystem II; NADPH, nicotinamide adenine dinucleotide phosphate).

In plant cells, chloroplasts, mitochondria, peroxisomes, plasma membrane, and cell wall are the major locations of ROS generation [38,39]. Therefore, compartmental ROS generation amounts to its overall production in plants (Figure 3) [6,40].

Chloroplasts are the prime sites for ROS production (30–100 times higher than mitochondria), depending on the interaction of chl and light, where triplet chl and ETC of PS I and II are the main sources of ROS production [6,40,41]. In PS II under illumination, chl in light harvesting complex (PSII-LHC) becomes excited to high-energy singlet state (^1^Chl*; short-lived, ~10^−8^ s). A portion of this energy is transferred to P680 by photochemical quenching (pQ) for driving the photosynthetic ETC. However, if the absorbed energy exceeds the pQ capacity, the excess energy is dissipated as heat or fluorescence or via intersystem crossing forming ^3^Chl* (lower energy; longer half-life, ~10^−3^ s) [42]. The carotenoids present in the LHC (lutein and zeaxanthin) quench ^3^Chl* preventing transfer of energy to other molecules. If this ^3^Chl* is not efficiently quenched, it reacts with ^3^O_2_ released from splitting of H_2_O in oxygen-evolving complex (OEC) leading to the formation of ^1^O_2_ [43]. Moreover, in the PSII reaction center (RC), P680 absorbs light energy and becomes excited to singlet state (^1^P680*) pairing with pheophytin (Pheo), ^1^(P680 ^+^ Pheo^−^) and later, transfers an electron to the quinone (Q_A_), forming P680^+^ Q_A_^−^. In an adverse situation, if Q_A_ is previously reduced thus being unable to accept any further electrons, ^3^(P680 ^+^ Pheo^−^) recombines with P680 forming an excited state ^3^P680* [44]. In the PSII RC, two molecules of β-carotene are present, which are capable of quenching this high-energy ^3^P680*, but the distance between them is too large (more than the Van der Waal’s distance of 3.6 Å) and quenching remains unsuccessful leading to generation of ^1^O_2_ [45]. In addition, some abiotic stresses causing stomatal closure drop chloroplastic carbon dioxide (CO_2_) levels leading to overreduction of the ETC and enhance the probability of charge recombination between ^1^P680* and Q_A_^−^ in PS II, increasing ^1^O_2_ production [46]. On the other hand, ^1^O_2_ is not produced at PS I [47], instead, O_2_^•−^ can be produced by Mehler reaction and later converted into H_2_O_2_ by SOD [48]. Later on, metal ions such as Fe^2+^ converts both O_2_^•−^ and H_2_O_2_ to highly stable ^•^OH [6]. In the nongreen plant parts, especially in roots, mitochondria are the main source of ROS production, where electron leakage from both complex I and III of ETC produces O_2_^•−^, which later catalyzed into H_2_O_2_ by Mn-SOD and CuZn-SOD [6,48]. In peroxisomes, glycolate oxidase (GOX) is the main source of ROS production [49]. Moreover, xanthine oxidase (XOD) activity can produce O_2_^•−^ and uric acid in peroxisomal matrix, which further dismutates to H_2_O_2_ by SOD and urate oxidase (UO), respectively [50,51,52]. Besides β-oxidation of fatty acids, O_2_^•−^ disproportionation and flavin oxidase activity could also produce H_2_O_2_ in peroxisomes [49,53]. In addition, polyamine oxidase, copper amine oxidase, sulfite oxidase, and sarcosine oxidase enzyme activity also can generate H_2_O_2_ in peroxisome [54]. However, the enzyme MDHAR has been demonstrated in peroxisomes, which helps to scavenge H_2_O_2_ by AsA-GSH cycle and regenerate AsA [55,56]. In the apoplast, NADPH oxidase, class III POX, amine and germin-like oxalate oxidases, quinine reductase, and lipoxygenases (LOX) contribute in ROS generation [11,57]. In the cell wall, POX, amine oxidases, and LOX activity are the potential source for ROS in the cell wall [7,58]. Furthermore, NADPH oxidase and quinone reductase guided the ROS production in the plasma membrane [58,59]. Apart from these prime sites, cytochrome (Cyt) P450 produces O_2_^•−^ in the endoplasmic reticulum. In this process, a radical intermediate (Cyt P450R−) is formed first by the reaction between Cyt P450 and organic substrate (RH) reduced by a flavoprotein. The resulted intermediate further reacts with ^3^O_2_ forming a radical oxygenated complex Cyt P450-ROO^−^, which is finally reduced by Cyt b or spontaneously decomposed to release O_2_^•−^ [58]. Fatty acid oxidation, as well as GOX and UO activities, produce O_2_^•−^ and H_2_O_2_ in glyoxysomes [60]. Moreover, both XOD and aldehyde oxidase (AO) are potentially involved in cytosolic ROS production [61].

## 4. Oxidative Stress in Plants and Downstream Implications

**Figure 4:**
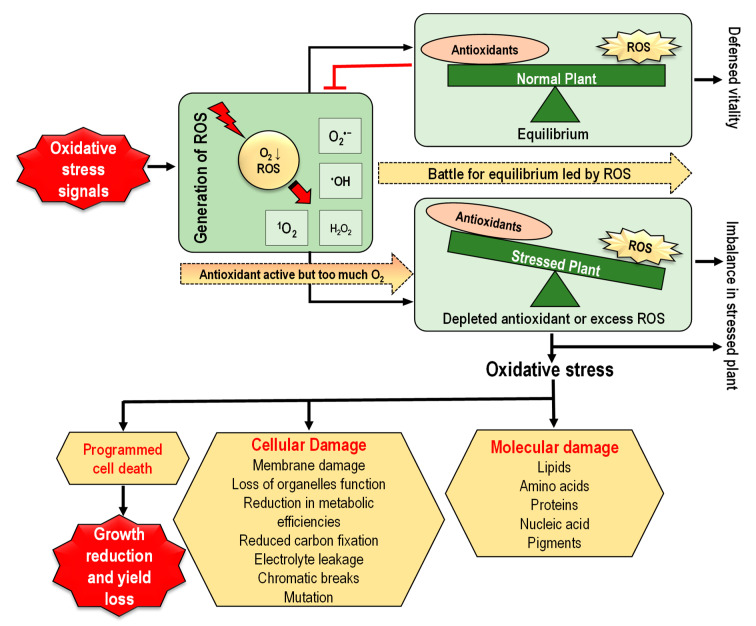
Oxidative stress in plants and its consequences (ROS, reactive oxygen species; ^1^O_2_, singlet oxygen; O_2_^•^^−^, superoxide anion; H_2_O_2_, hydrogen peroxide; ^•^OH, hydroxyl radical).

Redox reactions (transfer of electrons between a donor and an acceptor) are very common in living organisms, which is responsible for the production of ROS [62]. In plant cells, redox homeostasis is developed in consequence of the equilibrium between the generation of ROS and the functioning of the antioxidant enzymes where efficient defense system in plants keeps the proper balance between ROS generation and elimination [63]. A basal level of ROS, which is maintained above cytostatic or below cytotoxic concentration is, therefore, indispensable for proper ROS or redox signaling in cells, and this level is maintained by the balance between ROS production and ROS scavenging [4,11]. Therefore, scientists used the term “redox biology” to refer to ROS as signaling molecules to control and uphold the usual physiological activities of plants [11,64,65]. Redox signaling has been discerned as the equilibrium between low levels of ROS functioning as signals to activate signaling cascades that adjust usual plant functions and high levels of ROS causing oxidative cellular damage [62]. Therefore, a steady balance between ROS generation and ROS scavenging systems is strongly synchronized over time and space, working together with the cellular redox-sensitive components to shape and finely adapt downstream signaling procedures in a cell-specific and context-specific approach [66,67]. However, any disturbance in the equilibrium of ROS generation and ROS scavenging by antioxidants leads to ROS overaccumulation resulting in oxidative stress under various abiotic stress conditions [9]. Oxidative stress causes lipid peroxidation, damages nucleic acids and proteins, and alters carbohydrate metabolism, resulting in cell dysfunction and death (Figure 4) [4,68].

## 5. Oxidative Stress under Abiotic Stress

Plants are sessile organisms that normally grow under field conditions. Therefore, in most regions of the world, they face excess light (sunny hours) during the hot season. Besides, different environmental/abiotic stresses generated due to anthropogenic activities and harsh climate changes are contributing in inducing oxidative stress through overgeneration of ROS. It is well established that chloroplasts, mitochondria, peroxisomes, apoplast, and plasma membranes are the primary sites of cellular ROS generation but chloroplasts are the leading sites for ROS production [6,40]. Most of the abiotic stresses reduce the availability of CO_2_ and hinder carbon fixation and contribute to successive reduction of molecular oxygen, which yields excess ROS and impairs the performance of chloroplasts, thus disturbing photosynthetic processes [8]. However, ROS generation greatly varies with plant species, genotypes, stress tolerance level, and duration of stress exposure (Table 1).

### 5.1. Oxidative Stress under Salinity

Salinity affects plants by imposing various complications such as ion toxicity, osmotic stress, nutritional deficiency, and genotoxicity, resulting in ROS overproduction and oxidative stress (Table 1) [69]. For instance, Rehman et al. [70] found a 2.5- and a 3-fold, increase in the production of H_2_O_2_ together with a 2- and a 3-fold increase in thiobarbituric acid reactive substances (TBARS) content under 100 and 200 mM sodium chloride (NaCl) stress, respectively, compared with control depicting salt-induced oxidative stress condition. It is also reported that the oxidative stress varies among the plant tissues under salt stress. For instance, it was reported that root tissues suffered most from salinity-induced oxidative stress, followed by mature and young leaves. Recently, Cheng et al. [71] reported that the total ROS content, lipid peroxidation, and electrolyte leakage (EL) in rice root tissues were two times higher under salt stress compared with the control. In another study, Ahanger et al. [72] reported an overaccumulation of O_2_^•−^ and H_2_O_2_ (by 157% and 176%, respectively) together with increased malondialdehyde (MDA, by 94%) content and EL (by 158%) confirming salinity (100 mM NaCl)-induced oxidative stress in tomato. Similarly, both MDA and EL were increased by 2-fold due to salt stress (0.4%) in sweet peppers [73], while a 2-fold increase in H_2_O_2_, MDA, EL, and O_2_^•−^ content was found to be caused in mung bean following 100 mM NaCl exposure [74]. Moreover, increased H_2_O_2_ (by 50%) and MDA (by 25%) content were noted in maize plants under salt stress (120 mM NaCl) compared with controls [75]. The extent of oxidative stress varied among genotypes within a species. Lalarukh and Shahbaz [76] exposed two sunflower genotypes (FH-572 and FH-621) to salt stress (120 mM NaCl) and observed that H_2_O_2_ content increased (by 78%) in FH-572, while decreasing (by 20%) in FH-621, indicating FH-621 as being more salt stress tolerant. In a similar study, Tariq and Shahbaz [77] evaluated two sesame genotypes (TS-5 and TH-6) against salt stress (70 mM NaCl) and concluded that TS-5 showed comparatively better salt tolerance than TS-6. Similarly, Mhadhbi et al. [78] showed a genotype-dependent correlation between salinity tolerance and cellular damage indicators such as MDA and H_2_O_2_ content in *Medicago truncatula* genotypes under salt stress conditions. Interestingly, *Ailanthus altissima* plants growing under 150 mM NaCl had upregulated antioxidant enzymatic activities and no significant difference in H_2_O_2_ content compared with control plants, suggesting a link between the antioxidant defense apparatus and their increased invasiveness in adverse environments [79]. From these examples, it is evident that plants have differential responses towards salt-induced oxidative stress conditions.

### 5.2. Oxidative Stress under Water Deficit and Simulated Drought

Drought provokes stomatal closure, reduced CO_2_ entry, and impaired photosynthetic rate, as well as imbalance in the light harvest and utilization and altered photochemistry in chloroplasts, causing ROS overproduction [4,80]. Moreover, protein and membrane denaturation from photorespiration, inactivation of TCA cycle enzymes, and reduced carboxylation efficiency during drought are also linked with ROS overproduction [81]. Additionally, a lower NADP^+^ regeneration causes greater reduction of ETC, higher EL under drought stress, ultimately resulting in excess ROS metabolism and oxidative stress [82,83]. Many studies have reported drought-induced ROS overproduction and oxidative stress in numerous plant species (Table 1). Abideen et al. [84] grew *Phragmites karka* under drought conditions by maintaining 40% water holding capacity for 35 d in a plastic tube and found 22% increase of MDA content. Under similar conditions, Campos et al. [85] recorded higher MDA content in *Coffea arabica* L. after 20 d. Saha et al. [86] created drought conditions for rice plants by withdrawing irrigation for 8 d and found that in contrast to control, drought stress increased O_2_^•−^, H_2_O_2_, and MDA content by 1.8-, 2.1-, and 1.66-fold, respectively. Severe drought stress (75% water deficit condition) in finger millet plants considerably increased EL and H_2_O_2_ content [87]. In another study, Malhotra et al. [88] withheld irrigation in tomato plant for 6 d, which resulted in an increase of MDA content as well as 39% augmentation of EL. Hasanuzzaman et al. [89] and [90] investigated the effect of hyperosmotic stress (10% and 20% polyethylene glycol; PEG) on *Brassica napus* L. cv. Bina Sharisha-3 and found that both MDA and H_2_O_2_ increased under stress conditions. Similarly, hyperosmotic stress (5% PEG, 48 h) induced higher accumulation of H_2_O_2_ and O_2_^•^^−^ with enhanced membrane peroxidation and LOX activity in *Vigna radiata* L. cv. BARI Mung-2 [91]. Abbas et al. [92] observed higher TBARS, EL, and H_2_O_2_ contents in wheat grown under water deficit condition (70% field capacity; FC). A similar increase in O_2_^•−^, H_2_O_2_, and MDA content was observed in *Oryza sativa* L. var. *japonica* cv. Nipponbare grown under 20% PEG-induced hyperosmotic stress [93]. Rezayian et al. [94] observed significantly increased MDA, H_2_O_2_ content, and LOX activity in 15% PEG-stressed *Glycine max* plants compared with control samples. Rady et al. [95] exposed *Solanum lycopersicum* L. cv. Login 935 plants to drought stress (60% FC, 20 d) and observed increased O_2_^•−^, H_2_O_2_, and MDA contents by 75%, 37%, and 83%, respectively. In agreement with this, Filippou et al. [96] recorded significantly increased MDA and H_2_O_2_ content in *M. truncatula* plants under severe drought stress conditions, while parameters were reversed to prestress conditions following rewatering. The extent of drought severity also depends on the genotypic capability to cope with oxidative stress. Kusvuran and Dasgan [97] compared two *Phaseolus vulgaris* genotypes (Bn-150 (drought-tolerant) and Bn-16 (drought-sensitive)) under drought (50% FC, 14 d), where Bn-16 exhibited 2-fold greater MDA content than Bn-150. Moreover, O_2_^•−^, H_2_O_2_, and ^•^OH were also found to be higher in Bn-16.

### 5.3. Oxidative Stress under Metals/Metalloids Toxicity

Metals/metalloids generate ROS in plant cells by disrupting the chloroplastic and mitochondrial electron transfer activities as well as peroxisomal oxidative metabolism. A number of studies demonstrated toxic metals-/metalloids-induced overproduction of ROS and subsequent oxidative damage in different plants (Table 1). A remarkable increase in lipid peroxidation along with the higher accumulation of H_2_O_2_ was observed in *O. sativa* seedlings grown under nickel (Ni; 0.25 and 0.5 mM NiSO_4_, 72 h) toxicity [98]. El-Amier et al. [99] also reported similar results with lower levels of Ni (100 µM Ni as NiCl_2_) in *Pisum sativum*. On the other hand, cadmium (Cd) stress has been shown to increase MDA, H_2_O_2_, and O_2_^•–^ levels in different crops [100,101,102]. For example, Cd stress (100 µM CdCl_2_) resulted in increased MDA and H_2_O_2_ in *Arabidopsis thaliana* [103] and *Cucumis sativus* seedlings [104]. In a recent study, Ahanger et al. [105] reported that lipid peroxidation, EL, H_2_O_2_, and O_2_^•−^ contents as well as LOX activity were markedly increased in *V. angularis* seedlings under Cd stress (100 µM CdCl_2_, 20 d), while a similar increase in EL, H_2_O_2_ and TBARS contents was recorded in *Mentha arvensis* under Cd stress (50 µM CdCl_2_, 100 d) [106]. Hasanuzzaman et al. [107] demonstrated higher MDA, H_2_O_2_, and O_2_^•−^ content in wheat plants subjected to lead (Pb) stress (0.5 and 1.0 mM Pb(NO_3_)_2_) compared with control. The nonredox metalloid arsenic (As) has also been reported to induce oxidative stress. *Cajanus cajan* seedlings exposed to As (10 µM As as Na_3_AsO_4_, 5 d) had significantly higher H_2_O_2_, O_2_^•−^, and MDA content, as well as 4-fold increased LOX activity compared with unstressed plants [108]. Such an increase in H_2_O_2_ and TBARS contents was also evident in two different cultivars of *Chenopodium quinoa* under higher levels of As (150 and 300 µM Na_3_AsO_4_, 35 d) stress [109]. Interestingly, significant cellular damage in the form of increased MDA and H_2_O_2_ content was also observed in basil plants growing under copper (Cu) stress (1000 ppm), although no significant increase in MDA content was observed in plants growing under milder Cu stress conditions (500 ppm) [110].

### 5.4. Oxidative Stress under High Temperature

High temperature (HT) leads to the overproduction of ROS, resulting in altered cellular metabolism, inactivated oxygen-evolving complex and increased lipid peroxidation, membrane damage, and nicking of DNA, and further, it drives to cell death (Table 1) [111]. High temperature (35 °C/32 °C day/night) blocked PSII reaction center and electron flow, reduced quantum efficiency (Fv/Fm), and downregulated PSII photochemistry in two rice cultivars (IR64 and Huanghuazhan) [112]. Ding et al. [113] reported a 79.9% increase in O_2_^•−^ content cucumber (*C. sativus* L.) seedling due to HT stress (35 °C). In tobacco (*Nicotiana tabacum* cv. Bright-Yellow 2), HT (50 °C) increased 50% of O_2_^•−^ content over a period of 5 min creating oxidative stress. Likewise, Djanaguiraman et al. [114] observed O_2_^•−^ content increased by 3.5-fold and 2.3-fold in pollen and pistils, respectively, in field-grown sorghum plants under HT stress, but Liu et al. [93] did not observe any significant change in O_2_^•−^ and MDA content under HT stress (38 °C, 5 d), in spite of H_2_O_2_ increased by 1.27-fold in rice seeds compared with control samples.

### 5.5. Oxidative Stress under Low Temperature

Low temperature (LT) causes overproduction of ROS in plants through degradation of membrane fluidity, inhibiting photosynthetic apparatus activity, and imbalanced ROS detoxification, which lead to lipid peroxidation and EL (Table 1) [115]. Han et al. [116] showed increased MDA (by 180%) and EL (by 49%) contents in cold-stressed (12 °C, 6 d) 14-d-old rice seedlings. Similarly, Liu et al. [117] treated a cold-sensitive *S. lycopersicum* (Jinpeng No. 1) genotype with LT stress (15 °C/8 °C day/night; 24 and 48 h), leading to significantly higher MDA and H_2_O_2_ content compared with controls. Similarly, Xue et al. [118] evaluated wild-type (WT) and transgenic (G-1 and G-2) *Ammopiptanthus mongolicus* for LT stress tolerance in a controlled system (4 °C for first 24 h, 0 °C for next 12 h, and −6 °C for last 12 h) and found that WT plants accumulate higher levels of H_2_O_2_ compared with transgenic plants (detected through 3,3′-diaminobenzidine staining), indicating oxidative stress under LT stress.

### 5.6. Oxidative Stress under Flooding

Flooding or waterlogging (WL)-induced hypoxic or anoxic conditions generate toxic compounds that impair plant metabolism resulting in ROS overgeneration and oxidative damages (Table 1) [119]. Zhang et al. [120] also experimented with two *Sorghum bicolor* genotypes JN01 (WL-tolerant) and JN31 (WL-sensitive) and reported a remarkably higher accumulation of MDA in JN31 compared with JN01, at different duration (6, 9, and 12 d) of WL treatment. Anee et al. [121] studied the WL-sensitive *Sesamum indicum* L. cv. BARI Til-4 under different durations (2, 4, 6, and 8 d) of WL stress and reported that MDA and H_2_O_2_ increased in a duration-dependent manner. Similar enhancement of these oxidative stress markers was also observed in *S. lycopersicum* [122]. However, an Antarctic plant, named *Deschampsia antarctica*, also produced higher MDA and H_2_O_2_ when exposed to WL condition for 7 d [123].

## 6. Overview of Plant Antioxidant Defense System

**Figure 5:**
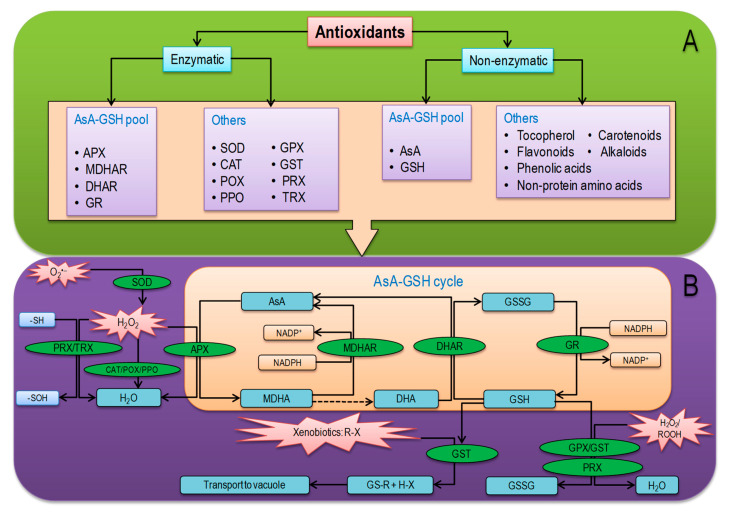
Overview of plant antioxidant defense system: (**A**) types of antioxidants and (**B**) combined mechanisms of enzymatic and nonenzymatic antioxidants. See the text for a more detailed description. APX, ascorbate peroxidase; AsA, ascorbate; CAT, catalase; DHA, dehydroascorbate; DHAR, dehydroascorbate reductase; GPX, glutathione peroxidase; GR, glutathione reductase; GSH, reduced glutathione; GSSG, oxidized glutathione; GST, glutathione *S*-transferase; H_2_O_2,_ hydrogen peroxide; MDHA, monodehydroascorbate; MDHAR, monodehydroascorbate reductase; NADPH, nicotinamide adenine dinucleotide phosphate; O_2_^•^^−^, superoxide anion; POX, peroxidases; PRX, peroxiredoxins; R, aliphatic, aromatic, or heterocyclic group; ROOH, hydroperoxides; –SH, thiolate; SOD, superoxide dismutase; –SOH, sulfenic acid; TRX, thioredoxin; X, sulfate, nitrite, or halide group.

Antioxidants directly or indirectly scavenge ROS and/or control ROS production [138]. The antioxidant defense system consists of low-molecular-weight nonenzymatic antioxidants and some antioxidant enzymes [4]. The nonenzymatic antioxidants such as AsA, GSH, α-tocopherol, phenolic compounds (PhOH), flavonoids, alkaloids, and nonprotein amino acids work in a coordinated fashion with antioxidant enzymes such as SOD, CAT, POX, polyphenol oxidase (PPO), APX, MDHAR, DHAR, GR, GPX, GST, TRX, and PRX in order to inhibit overproduction of ROS (Figure 5) [139,140]. The catalytic reaction of enzymatic and nonenzymatic antioxidants and the reaction sites in cellular organ is represented in Table 2. In plants, the enzyme SOD is directly related to stress, which initiates the first line of defense, converting O_2_^•−^ into H_2_O_2_ (Table 2) [141,142]. This generated H_2_O_2_ can be further converted into H_2_O by the enzymes CAT, APX, GPX, or catalyzed in the AsA-GSH cycle. In plant cell, the AsA-GSH cycle or Asada—Halliwell cycle is the major antioxidant defense pathway to detoxify H_2_O_2_, which consist nonenzymatic antioxidants AsA and GSH as well as four important enzymes APX, MDHAR, DHAR, and GR. In the antioxidant defense system, a key role is performed by the AsA-GSH cycle to minimize H_2_O_2_ and redox homeostasis [4,143]. In addition, GPX and GST are also vital enzymes for the detoxification of H_2_O_2_ and xenobiotics (Figure 5) [144]. Among nonenzymatic antioxidants, AsA and GSH are the most abundant soluble antioxidants in higher plants [145], those play a vital role as electron donors and scavenge ROS directly through AsA-GSH cycle [4]. Moreover, beta-carotene reacts with ^•^OH, O_2_^•−^, and ROO^•^ radicals resulting in reduced cellular ROS concentrations [146].

### 6.1. Nonenzymatic Antioxidants

Ascorbate plays a significant role in AsA-GSH cycle to scavenge ROS through its capacity to donate electrons and remain stable due to electron delocalization that results from the resonance between two forms [4]. Many phytohormone biosynthesis pathways are regulated by AsA. Moreover, AsA regenerates α-tocopherol (vitamin E) from tocopheroxyl radical or by scavenging of ^•^OH and O_2_^•−^ [147,148]. Contrarily, another vital component of the antioxidant defense system, GSH, plays a significant role in the regulation of AsA-GSH cycle for scavenging cellular ROS and redox homeostasis [4]. Tocopherol protects the chloroplast and maintains photosynthesis by scavenging ROS, mainly ^1^O_2_ and ^•^OH [149]. Carotenoids constitute another important class of antioxidant molecules, which are known to scavenge harmful free radicals, as well as to protect light-harvesting complex proteins and thylakoid membrane stability [3,150]. Low-molecular-weight compound flavonoids and, especially, dihydroxy B-ring-substituted flavones and flavonols have great potential to scavenge free radicals and reduce cell damage from lipid peroxidation [151,152,153]. Moreover, abiotic stresses upregulated the expression of genes related to flavonoids biosynthesis and activate antioxidant defense mechanisms [3]. The antioxidant phenolic acids are mainly composed of hydroxybenzoic and hydroxycinnamic acids, those show antioxidant activity as chelators and scavengers of free radicals, especially O_2_^•−^, ^•^OH, ROO^•^, and ONOO^–^ [138]. Alkaloids also have antioxidant ability as free radical scavengers and inhibit H_2_O_2_-induced oxidation [154]. Furthermore, nonprotein amino acids (gamma-aminobutyric acid, ornithine, and citrulline) are also considered as effective nonenzymatic antioxidant [155].

### 6.2. Antioxidant Enzymes

Superoxide dismutase (SOD; EC 1.15.1.1), categorized into three main types—Cu/Zn-SOD, Fe-SOD, and Mn-SOD, leads the frontline defense in the antioxidant defense system by dismutating O_2_^•−^ into H_2_O_2_ and reducing the possibility of ^•^OH formation [156]. In the antioxidant defense system, catalase (CAT; EC 1.11.1.6) is a tetrameric heme-containing enzyme for ROS detoxification, which converts 26 million H_2_O_2_ molecules into H_2_O in 1 minute [3]. Peroxidase (EC. 1.11.1.7) mainly oxidizes PhOH for producing phenoxyl radical (PhO^•^) more commonly referred to Q_A_, where H_2_O_2_ accepts electron and is converted to H_2_O. In the absence of AsA, PhO^•^ cross-reacts producing suberin, lignin, and quinines, but in the presence of AsA, PhO^•^ reacts with AsA generating monodehydroascorbate (MDHA) and, subsequently, DHA (Figure 5 and Table 2) [157].

Polyphenol oxidase (EC 1.14.18.1) mostly found in thylakoid membrane of chloroplast can influence photosynthesis directly. The enzyme polyphenol oxidase could also interact with peroxidase, or water–water cycle to facilitate ROS scavenging. PPO oxidizes PhOH to Q_A_ and H_2_O by using available O_2_ [158]. In plant cells, AsA-dependent APX (EC 1.11.1.1) occurs in different isoforms (cytosolic APX (cAPX), mitochondrial APX (mtAPX), chloroplastic APX (chlAPX; APX is the only enzyme capable of scavenging H_2_O_2_ in chloroplast since CAT is not present), and peroxisomal/glyoxysomal APX (mAPX; including)) and are H_2_O_2_ scavengers, which participates in AsA-GSH cycle producing monodehydroascorbate (MDHA) [159]. The produced MDHA is converted to AsA by a NADPH-dependent flavin adenine dinucleotide enzyme—MDHAR (EC 1.6.5.4)—found as two isoforms in various cellular locations [4]. Monodehydroascorbate reductase containing a thiol group regenerates AsA by phenoxyl radical reduction [160]. Monodehydroascorbate is further reduced into DHA nonenzymatically, which is then recycled to AsA by the activity of GSH-dependent DHAR (EC1.8.5.1) activity [160]. In this reaction, GSH is oxidized to GSSG that is further reduced to GSH by NADPH-dependent GR (EC 1.6.4.2) enzyme, which is also vital for the regulation of redox homeostasis [161]. Beyond, GPX (EC 1.11.1.9) is a member of nonheme containing POX family antioxidant enzyme having a highly reactive thiol group, which utilizes GSH and TRXs to scavenge H_2_O_2_, reducing lipids, and organic hydroperoxides [162]. In line, GST (EC 2.5.1.18) conjugates GSH and electrophilic substrates, in its active sites, thus metabolizing xenobiotics (especially, herbicides and pharmaceutically active compounds) and transport them into vacuoles [163,164]. It is also involved in peroxide breakdown, hormone biosynthesis, and stress signaling as well as accelerating GPX activity [165,166]. Moreover, TRX (EC 1.8.1.9), having different isoforms (*f*, *m*, *h*, *s*, *o*, *x*, *y,* and *z*), contain an active redox site (WCG/PPC), which reduces disulfide bonds into dithiol by H_2_O_2_ and regulate the target proteins faster than GSH or dithiothreitol [167]. Among the isoforms, TRX*x* and TRX*y* can regulate redox homeostasis in chloroplast by reducing 2-Cysteine (Cys) PRX, whereas in mitochondria, TRX*o1* participates with PRX and sulfiredoxin to activate antioxidant defense [168]. Another antioxidant enzyme, the thiol-based PRX (EC 1.11. 1.15), exhibits POX-like activity neutralizing peroxides (H_2_O_2_ and ROOH) in the cytosol, chloroplasts, mitochondria, and nucleus of plant cells [169,170]. The PRXs are thiol-dependent (GSH or any other thiol), playing a vital role in ROS regulation due to the capability of reducing various organic and inorganic peroxides (Figure 5 and Table 2) [171].

## 7. Antioxidant Defense in Plants under Abiotic Stress: Recent Approaches

Plants activate their antioxidant defense system to mitigate the adverse effects of oxidative stress. However, antioxidant defense capacity varies among plant species and genotypes, as well as stress types and duration (Table 3). Moreover, different approaches to enhance plant antioxidant defense have also been revealed (Table 3).

### 7.1. Antioxidant Defense in Plants under Salinity

Regulation of antioxidant machinery ameliorates the effects of salt stress in plants, as reported in many plant studies (Table 3). Researchers have reported that differential activities of antioxidant enzymes vary according to salinity extent, exposure time, and the developmental stages of plants [172,173]. For instance, Vighi et al. [174] observed differential response in salt-tolerant (BRS Bojuru) rice cultivar compared with salt-sensitive (BRS Pampa) one and concluded that *OsAPX3*, *OsGR2*, *OsGR3*, and *OsSOD3-Cu/Zn* genes were the basic differential markers between tolerant and sensitive rice genotypes. In another study, Zeeshan et al. [175] compared wheat (salt-tolerant cv. Suntop and -sensitive Sunmate) and barley (salt-tolerant cv. CM72) cultivars and concluded that higher activities of antioxidants (SOD, peroxidase; POD, APX, GR, and CAT) are strongly correlated with the higher salt tolerance depicting a clear role of antioxidant activities in mitigation of salt-induced oxidative stress. Similarly, Alzahrani et al. [124] found increased SOD, CAT, GR, and AsA levels in faba bean genotypes, when H_2_O_2_ increased above 90% under salinity stress, confirming the regulation of antioxidant response under salt stress and its mitigation. The regulation of antioxidant activities through the use of either chemical or natural protectants under salt stress has also confirmed the role of plant antioxidant machinery in ameliorating stresses such as salinity [176,177,178]. For instance, Alsahli et al. [179] found that a 2-fold increase in SOD, CAT, and APX activity decreased 3-fold H_2_O_2_ in salt-stressed wheat by salicylic acid (SA) application compared with untreated control plants. Similarly, the combined application of jasmonic acid (JA) and humic acid also increased APX activity, resulting in salinity tolerance in sorghum [180], while exogenous application of polyamines regulated sour orange antioxidant responses under salinity stress conditions [181]. Nitrogen supplementation is also reported to increase the antioxidant (SOD, CAT, APX, GR, MDHAR, DHAR activities and the biosynthesis of AsA and GSH) levels with declining 2.5-fold H_2_O_2_ and 1.7-fold O_2_^•−^ generation in wheat under 100 mM NaCl stress [182]. Moreover, silicon (Si) supplementation also increased antioxidant activities and decreased ROS, MDA, and EL levels in mung bean under salinity [74]. Chung et al. [183] reported Si-induced upregulation of antioxidant enzyme genes *GmCAT1* (by 3-fold)*, GmCAT2* (by 4-fold)*,* and *GmAPX1* (by 8-fold), leading to salt stress tolerance in soybean after 6 h of stress exposure. Similar transcriptional regulation of antioxidant enzyme transcript levels (*cAPX, CAT, GR,* and *MnSOD*) decreased 0.4-fold H_2_O_2_ and 3.9-fold NO in hydrogen sulfide (H_2_S)-primed strawberry plants under NaCl stress in a hydroponic setup [184]. Santander et al. [185] reported that arbuscular mycorrhizae-induced increased SOD, CAT, and APX activities in 40 and 80 mM NaCl-stressed cucumber. Moreover, *Moringa oleifera* leaf extract (6%) or *Glycyrrhiza glabra* root extract mitigates salt stress by upregulating antioxidants in wheat and bean (*P. vulgaris*) [186,187]. Finally, supplementation of fungicidal compound penconazole (15 mg L^−1^) regulated SOD, CAT, POX, and PPO activity to mitigate the negative impact of salinity in sesame (*S. indicum*) [188]. From the above-discussed examples, the overall influence of plant antioxidant system in the mitigation of salt stress and associated oxidative stress conditions is clear and an immensely important factor to be aware of.

### 7.2. Antioxidant Defense in Plants under Water Deficit and Simulated Drought

Activating antioxidant defense as an adaptive mechanism against drought stress was reported in different plants (Table 3) [83,89,90]. Nahar et al. [91] demonstrated decreased AsA/DHA and GSH/GSSG ratio with increased APX, GR, GPX, and GST activities in drought-exposed *V. radiate* seedlings compared with control, which contributed in drought-induced oxidative damage tolerance. Akram et al. [189] compared the performances of two canola cultivars *B. napus* (cv. Dunkeld and Cyclone) exposed to water deficit condition (60% FC, 21 d) and found increased total phenolic contents as well as upregulated CAT and POD activities in both cultivars. When studying two *Sorghum bicolor* L. cultivars (M-81E (tolerant) and Roma (sensitive)), Guo et al. [190] found that drought stress increased 28.9% and 54.9% H_2_O_2_ in M-81E and Roma, respectively, relative to control, when SOD activity increased 1.6 and 1.1 times and APX activity increased 1.7 and 0.9 times, respectively, to improve drought tolerance. Yet, inhibited CAT activity but enhanced GPX activity was found under drought stress (irrigation stopped at 10 days after sowing, 11 d) in *Triticum aestivum* cv. Sakha-94 [191]. Meanwhile, a comparative study with *Zea mays* cv. Xida 889 and Xida 319 subjected to drought (50% FC, 15 d) reported that GSH content increased by 17% and 28% in Xida 319 and Xida 889, respectively, compared with the well-watered condition [150]. In addition, Rady et al. [95] observed higher H_2_O_2_ (26.2%) and O_2_^•−^ (51%) generation with enhanced SOD, CAT, and APX activities by 110%, 66%, and 77%, respectively, as well as significantly increased AsA, GSH, and α-tocopherol content in *S. lycopersicum* cv. Login 935 exposed to drought stress (60% FC, 20 d), which indicates increased antioxidant capacity to tolerate drought-induced oxidative stress. Improved tolerance against drought stress through the regulation of the antioxidant apparatus has also been shown in a number of chemical priming approaches, such as that of Antoniou et al. [130] where pretreatment of *M. sativa* plants with melatonin resulted in increased CAT activity and lowered H_2_O_2_ content compared with unprimed, drought-stressed plants. Similarly, the employment of nitric oxide and hydrogen sulfide aspirin (NOSH-aspirin) leads to improved performance in *M. sativa* plants under severe drought stress through the regulation of CAT and SOD activity, as well as *cAPX, Cu/ZnSOD,* and *FeSOD* transcripts [192].

### 7.3. Antioxidant Defense in Plants under Toxic Metals/Metalloids

Metals/metalloids toxicity tolerance is positively correlated with improved antioxidant activities for ROS detoxification and metal chelation (Table 3) [68,193]. Among major antioxidants, GST assists GSH to reduce metals/metalloids toxicity by conjugating with them [166]. Additionally, GSH works as a cytosolic precursor of phytochelatins (PC), which bind the metals and facilitates the compound transport into cell vacuole by catalyzing the shuttle of metal ions and other xenobiotics [9,194]. Movement of cytosolic metals/metalloids ions into the vacuole in inert form reduces cellular toxicity [68]. Moreover, both GST and GSH contribute in the accumulation of some flavonoids (anthocyanin), which also act as metal binder and may use the same pathway to be accumulated into the vacuole [195,196]. Hasanuzzaman et al. [98] observed an enhancement in both the GSH and GSSG contents in *O. sativa* seedlings under Ni stress (0.25 and 0.5 mM NiSO_4_^.^7H_2_O), among which GSH was further increased, but GSSG declined by exogenous Si (0.05 mM Na_2_SiO_3_) application signifying the role of Si in upregulating GSH. Moreover, Ni stress-induced enhancement of SOD, GPX, APX, MDHAR, DHAR, and GR activities, which was further upregulated by Si supplementation that helped to minimize Ni toxicity. Ahanger et al. [105] reported an increment in GSH and tocopherol content along with SOD, GST, and DHAR activities with higher H_2_O_2_ (61%) and O_2_^•−^ (47%) content in Cd-stressed (100 µM CdCl_2_, 20 d) *V. angularis* seedlings, whereas AsA levels and CAT activity declined. Contrarily, SOD, CAT, POX, and GR activities were upregulated with higher content of H_2_O_2_ (53.45% and 69.83%, respectively) under Cd stress (50 µM CdCl_2_, 100 d) in two *Mentha arvensis* (cv. Kosi and Kusha) genotypes pointing out the activation of an antioxidant defense system for conferring Cd toxicity tolerance [106]. The authors also reported a further upregulated antioxidant defense following application of gibberellic acid, triacontanol, or SA. Mahmud et al. [102] measured the AsA, DHA, GSH, and GSSG contents of *B. juncea* seedlings grown under Cd toxicity (0.5 and 1.0 mM CdCl_2_, 3 d) and found that AsA content along with CAT, MDHAR, DHAR, and GR activities declined in a dose-dependent manner, which was reversed by citric acid (CA, 0.5 and 1.0 mM) cotreatment. Moreover, CA cotreatment increased GSH content, SOD, APX, and GPX activities, and further assisted in reducing oxidative stress [102]. Lead (Pb) stress (1.0 mM Pb(NO_3_)_2_) resulted in lower AsA content as well as declined CAT, MDHAR, GR, and GPX activities with increasing H_2_O_2_ content by 41% and 95% at mild and severe stress, respectively, in wheat seedlings, which was inversely altered by 1.0 mM of GSH supplementation, thus demonstrating the effect of GSH in activating antioxidant defense system [107]. However, exogenous spermidine assisted in the restoration of AsA and GSH contents, as well as AsA/DHA and GSH/GSSG ratio, together with APX, DHAR, GR, and CAT activity, resulting in lower aluminum (Al; AlCl_3_ 0.5 mM, 48 and 72 h)-induced oxidative stress in *V. radiate* seedling [133].

### 7.4. Antioxidant Defense in Plants under High Temperature

Like other abiotic stress factors, the antioxidant defense mechanism is activated to cope with high temperature (HT) stress in plants (Table 3) [113,197], but overall antioxidant capacity differs between species as well as tolerant and sensitive genotypes [9]. According to Kumar et al. [149], the activity of APX and GR was significantly suppressed in sensitive chickpea genotypes (ICC14183 and ICC5912) with increasing almost 2-fold H_2_O_2_ under HT conditions in comparison with tolerant genotypes (ICCV07110 and ICCV92944). Liu et al. [93] reported decreased SOD and CAT activities with corresponding suppressed *OsSOD, OsCAT,* and *OsAPX2* expression, resulting in higher accumulation of H_2_O_2_ (1.27-fold) in germinating rice seeds under HT stress. Sarkar et al. [198] found elevated activity of CAT and POX in wheat genotypes under HT stress (30 °C). In another example, Zandalinas et al. [199] observed increased GSH and AsA content in Carrizo citrange along with enhanced SOD and CAT activity compared with Cleopatra mandarin under HT stress (40 °C). Furthermore, Sarwar et al. [134] pretreated cotton plants with H_2_O_2_ under HT stress and found increased SOD and CAT activity in comparison with unprimed, HT-stressed plants. Similar findings were reported by Christou et al. [20] who showed that strawberry plants pretreated with sodium hydrosulfide (NaHS) under HT stress (42 °C, 8 h) became more resilient than unprimed, stressed plants, and this was linked with the enhanced transcription of AsA (*GDH*) and GSH biosynthetic enzymes (*GS, GCS*), as well as enzymatic antioxidants (*cAPX, CAT, MnSOD,* and *GR*).

### 7.5. Antioxidant Defense in Plants under Low Temperature

Plants activate the antioxidant defense system to cope with low temperature (LT) stress as well (Table 3). A 3- and 2-fold increased Cu-ZnSOD and Fe-SOD activities, respectively, to a response of higher H_2_O_2_ and O_2_^•−^ production were reported in cucumber (*C. sativus* cv. Xinyan 4) seedling exposed to LT [15/8 °C day/night, 8 d] stress [200]. Moreover, significantly increased CAT activity was observed in *Cynodon dactylon*, *Capsella bursa pastoris,* and *Citrus reticulata*, during LT stress [201,202,203]. Contrarily, higher APX activity was observed in *Jatropha macrocarpa* as a response to high H_2_O_2_, which improved LT stress tolerance, whereas reduced APX activity (>6-fold) in *J. curcas* was linked with increased sensitivity under LT conditions [204]. Cheng et al. [205] experimented with *Citrullus lanatus* under LT stress (10/5 °C, 7 d) and observed the activation of the antioxidant defense system, where GSH/GSSG and AsA/DHA ratios increased significantly only a day after treatment compared with control samples. Similarly, Wang et al. [206] observed increased AsA and GSH levels as a response to higher H_2_O_2_ content in transgenic apple seedlings under LT stress (8 °C, 12 h). More recently, Han et al. [116] exposed 14-d-old rice seedlings to LT (12 °C, 6 d) stress and reported higher content of H_2_O_2_ and O_2_^•−^ accumulation increased SOD and CAT activity along with enhanced GSH/GSSG ratio.

### 7.6. Antioxidant Defense in Plants under Flooding

Several crop species exhibit their ability to survive under the flooded or WL condition for short or even longer durations by activating antioxidant defense systems (Table 3). Li et al. [207] exposed 18 maize genotypes to WL conditions. After 2 d of WL stress, 19–57% higher SOD activity was observed in 12 genotypes, 19.16–106.96% higher POD activity was found in 13 genotypes, while 26–57% higher CAT activity was found in only 9 genotypes. Lower AsA but increased GSH and GSSG content along with higher H_2_O_2_ content were observed in sesame seedling under WL stress in a time-dependent manner [121]. However, AsA-GSH cycle enzymes were not regulated in the same manner, showing significantly higher APX and MDHAR activity and lower DHAR and GR activity, during prolonged (8 d) WL stress [121]. Moreover, Park and Lee [123] recorded higher H_2_O_2_ (52%) accumulation increased 91% higher CAT activity compared with controls in the Antarctic plant *D. antarctica* exposed to WL (7 d) conditions.

## 8. Revisiting ROS Signaling in Plant Defense

Excess ROS are generated under abiotic stress owing to the disturbance of different metabolic functions and physiological disorders [5]. The antioxidant defense pathways such as the AsA-GSH pathway require energy in the form of NADPH, and once this energy is depleted, these pathways would be incapable of avoiding ROS toxicity [5,218]. However, the functions of ROS (especially H_2_O_2_) in plant responses to stresses came into the spotlight at the end of the 20th and the beginning of the 21st century. Few groups of scientists recognized H_2_O_2_ as a signaling molecule, which leads to acclimation processes and confers tolerance under different biotic and abiotic stresses [219,220]. Reactive oxygen species generated in the chloroplast during stress might divert electrons from the photosynthetic machinery preventing overload of the antenna and subsequent damage. Reactive oxygen species also protect mitochondria in a similar way [5,221]. Cell wall peroxidase might contribute to ROS generation towards signaling where H_2_O_2_ utilizes Ca^2+^ and MAPK pathway as a downstream signaling cascade. Moreover, plant hormones, especially ethylene (ET) and abscisic acid (ABA), are involved with stress responses via cross-talk with ROS and enhance stress tolerance, which confirms the dual role of ROS under stress condition [222]. Besides signal transduction and interaction with hormones, ROS can also regulate metabolic fluxes under abiotic stress conditions, which jointly control plant acclimation processes where redox reactions control transcription and translation of stress acclimation proteins and enzymes, ultimately protecting plant cells from damage [5,11]. Moreover, H_2_O_2_ modulates NO and Ca^2+^ signaling pathways, which control plant growth and development, as well as other cellular and physiological responses under diverse abiotic stresses [223,224]. As endogenous H_2_O_2_ is involved in increasing tolerance against abiotic stress, exogenous application of H_2_O_2_ is gaining increasing attention and has largely proved its efficacy [13,18,22,225]. In Table 4, we have listed some key reports dealing with the effect of H_2_O_2_ treatment under different abiotic stress conditions.

Moreover, ROS collaborate with RNS, RSS, and RCS under stress conditions and work jointly in signal transduction pathways [23,226]. Cellular antioxidant levels might be influenced to alter ROS generation and contribute to signaling [227]. On the other hand, RSS influence the manufacture, perception, and further signaling of ROS and RNS [226], while RCS act downstream of ROS as signal mediators under a variety of stress situations [228]. Therefore, the interacting role among ROS, RNS, RSS, and RCS is discussed in the following section.

## 9. Cross-Talk of Reactive Nitrogen, Sulfur, and Carbonyl Species with ROS

**Figure 6:**
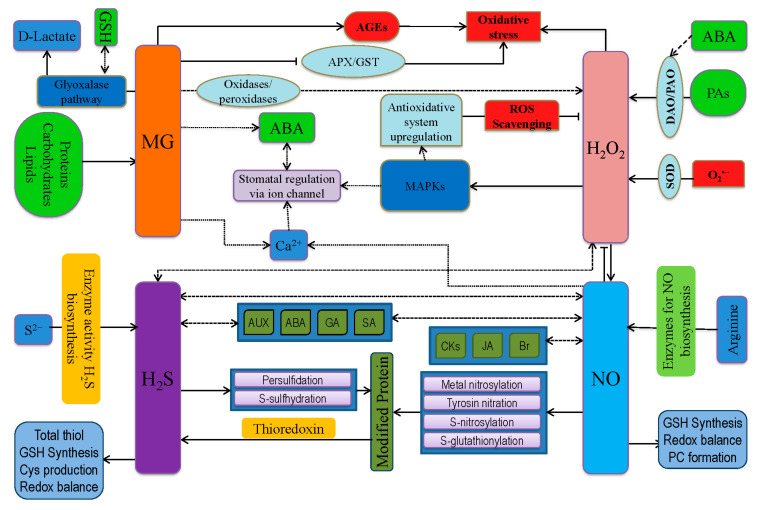
Cross-talk among vital ROS (H_2_O_2_), RNS (NO), RSS (H_2_S), and RCS (MG) in plant cells for oxidative stress and defense response in plants. APX, ascorbate peroxidase; AUX, auxin; ET, ethylene; ABA, abscisic acid; ROS, reactive oxygen species; GSH, reduced glutathione; JA, jasmonates, MAPKs, mitogen-activated protein kinases; SA, salicylic acid; AEGs, advanced glycation end products; PAs, polyamines; MG, methylglyoxal; NO, nitric oxide; H_2_S, hydrogen sulfide. Dotted lines represent activation/enhancement.

Apart from ROS, other reactive species are produced in plant cells during adverse environmental conditions, including RNS, RSS, and RCS (Figure 6) [146,226,232]. All these reactive species are involved in a molecular cross-talk and have a particular role in cellular signaling cascades [23]. Therefore, the following subsections discuss the intimate relationship among ROS, RNS, RSS, and RCS.

### 9.1. Interaction between RNS and ROS

Nitric oxide (NO) is considered to be the most important RNS in plants and is considered as one of three gasotransmitter molecules (Figure 6) [233,234]. During abiotic stress, ROS is overproduced, resulting in enhanced NO generation primarily by nitrate reductase (NR), indicating an interconnection between ROS and RNS [83,235]. As previously reported, NO generation increased by 8-fold in *Arabidopsis* following exogenous H_2_O_2_ application and subsequent NO accumulation activated antioxidant defense system, reduced ROS overgeneration, and reestablished redox balance [83,235]. Contrarily, H_2_O_2_ removal by antioxidants or NADPH oxidase inhibitor prevents NO production [236]. Likely, Ca^2+^-channel inhibitors (ned-19; 3-(2,3-dichlorophenyl)-1,1-dimethylurea and antimycin A) also inhibit H_2_O_2_-induced NO production [237]. Moreover, H_2_O_2_ is vital for ABA-mediated NO production [238]. Differently, ABA-induced H_2_O_2_ production is not NO dependent, which was confirmed by treating with NO donor (sodium nitroprusside, SNP), NO scavenger, and NO synthesis inhibitor. Therefore, H_2_O_2_ actively can modulate NO synthesis, via the NR activity [239]. Importantly, both H_2_O_2_ and NO play a vital role in signal transduction as well as phytotoxicity [240]. They also cross-react, generating ^•^OH, which is highly reactive.

H_2_O_2_ + NO → HNO_2_ + ^•^OH (11)

Although H_2_O_2_ is detoxified by CAT, APX, and GPX, a small amount might escape, which reacts with NO and generates damaging ^•^OH [240,241]. In the absence of metal ions, this reaction is one of the most important mechanisms for generating ^•^OH, providing new insights for ROS-induced tissue-specific oxidative damage as well as signal transduction guided by NO and/or H_2_O_2_ [240]. Under abiotic stress conditions, a number of ROS and RNS, e.g., ^1^O_2_ and ONOO^−^ might form, leading to oxidative damage [242]. On the other hand, O_2_^•−^ reacts with NO producing a powerful oxidant, peroxynitrite (ONOO^–^), which participates in the post-translational modification (PTM) of tyrosine nitration of proteins [243,244]. In peroxisomes, *S*-nitrosylation inhibits the functioning of glycolate oxidase, CAT, and it can regulate H_2_O_2_ levels at the cellular level [245], whereas, the generated ONOO^−^ molecule cause tyrosine nitration and nitrosative alteration in plants. In addition, the proteomic evaluation showed peroxisomal NADH-dependent hydroxypyruvate reductase, which is dysfunctioned by peroxynitrite through nitration [50]. Therefore, it could be concluded that the cross-talk among the ROS and RNS leads to several damaging or signaling episodes where many factors participate for building a complex network of ROS/RNS.

### 9.2. Interaction between RSS and ROS

Sulfur (S) is the fourth major essential plant nutrient and a structural component for secondary sulfur compounds, such as polysulfides, glucosinolates, PC, thiols, GSH, *S*-nitrosoglutathione (GSNO), and sulfolipids (Figure 6) [144,234]. Generally, RSS is sometimes called second-generation reactive species as they are formed from the reaction between S and ROS [246]. Interaction of ROS with thiols produces sulfenic acid (R-SOH), further to disulfides, which can donate electrons [246]. Again, R-SOH can undergo PTM (*S*-glutathionylation and S-cysteinylation), leading to protein stability and proper functioning [247]. Moreover, sulfide-*S*-oxides can be formed by the decomposition of *S*-nitrosothiols in the presence of high GSH concentration. This mechanism plays vital role in maintaining the redox balance of thiols as well as modulating *S*-proteins [248]. Meanwhile, the transformation of R-SOH to sulfinic acid to sulfonic acid is also possible by the oxidation via ROS [249]. H_2_S actively interacts with ROS to regulate the plasma membrane antiporter (Na^+^/H^+^) system [250]. Contrarily, H_2_S activates enzymatic antioxidants (SOD, CAT, and APX) and enhances GSH content, thus reducing oxidative damages [144,251]. Moreover, H_2_S is involved in production, perception, and further signal transduction of ROS as well as RNS [234]. As an integral part of the AsA-GSH cycle, redox ratio of GSH:GSSG is important for H_2_O_2_ scavenging, which is influenced by H_2_S [234,251]. Moreover, AsA content is also manipulated by H_2_S, thus maintaining proper ROS scavenging and acting as a protective molecule at lower cellular concentration [251]. Cysteine plays vital role at the chemical signaling junction of ROS and RSS, where both the molecule regulates signal by Cys-oxidation on proteins. Peroxidation of Cys-S by H_2_O_2_ produces the Cys-peroxide, Cys-SeOH [252], whereas persulfidation with H_2_S_2_ produces a Cys-persulfide [253]. In addition, Cys-persulfidation prior to protein synthesis has been described [254], but where these Cys-persulfides are targeted yet unidentified as well as the specific regulatory proteins remains to be determined.

### 9.3. Interaction between RCS and ROS

There is an intimate relationship between RCS and ROS. Reactive carbonyl species include mainly unsaturated aldehydes and ketones produced during lipid peroxidation, which mediate ROS signals. A dozen different RCS are reported to be produced from various membranes [255]. In addition, if RCS is exogenously added to the plants, a similar response as that induced by ROS could be obtained [255]. Considering these criteria, the damaging/signaling role of RCS has been reported to induce root injury, PCD, senescence of leaves and fruits, ABA-mediated stomatal closure, and root response to auxin. Thus, RCS act downstream of ROS as signal transducers during a variety of physiological situations [228]. On the other hand, RCS can modulate antioxidants (CAT, APX, and POD) and thus induce ROS overaccumulation and oxidative stress [256]. It has been demonstrated that RCS-induced ROS generation leads to ABA signaling suggesting ABA-induced ROS (H_2_O_2_) production in the guard cell, which increases RCS levels and modulates the signal for stomatal closure [257]. Among RCS, acrolein and 4-Hydroxy-2(E)-nonenal (HNE) formation is stimulated by ROS very early, but other RCS like crotonaldehyde, (E)-2-pentenal, and (E)-2-hexenal are also induced by ROS signals [258]. Furthermore, auxin signaling can induce ROS and RCS formation leading to lateral root initiation [228].

Kaur et al. [259] reported that the generation of methylglyoxal (MG) under stress could overaccumulate ROS directly or induce advanced glycation end products (AGEs) formation. Reports also suggested that increased O_2_^•−^ production is accelerated by MG [260]. Methylglyoxal also induced ABA or methyl jasmonates or NAD(P)H deficit dependent on stress signaling. Like ROS, RCS might modify Cys residues of proteins in a reversible way to regulate their activity, which would be effective at low levels. However, at higher levels, RCS can have deleterious effects on proteins since histidine and lysine residues can also react with RCS to form stable adducts and might mimic ROS signals potentially associated with regulating activities of proteins such as TRx, which can further regulate activities of other target proteins via redox regulation [256]. Reactive carbonyl species can also interact with zinc ion (Zn^2+^) and thus release Zn^2+^ from proteins affecting transcription factors (Zn finger proteins) as well as metabolic and defense enzymes [261]. Therefore, whether RCS could limit the activity of ZnSOD and other antioxidants could be a new area of research. In conclusion, ROS can increase RCS production, with RCS potentially interfering with the antioxidant defense system and exerting oxidative stress. Moreover, RCS can also modulate phytohormone biosynthesis and other signals, thus playing a vital role in constructing a complex ROS/RCS network. Therefore, deeper research should be done in this regard to open a new door of climate-smart crop production.

## 10. The Transgenic Approach in Enhancing Antioxidant Defense in Plants

In the past few decades, transgenic approaches have been widely used to improve plant health under oxidative stress. Thus, transgenic plants can be engineered to enhance stress tolerance and the activities of antioxidant enzymes. An overview of transgenic plants with enhanced activities of antioxidant defense systems under several stresses is presented in Table 5. Kiranmai et al. [262] isolated a *MuWRKY3* gene from drought-adapted horse gram and overexpressed it in groundnut. Transgenic plants showed lower MDA, H_2_O_2_, and O_2_^•−^ contents and enhanced the activities of SOD by 3 to 5-folds and APX by 3 to 7-folds, resulting in increased drought tolerance. Overexpression of *MdATG18a* in apple enhanced tolerance to drought stress and increased the activities of CAT and POD by 2-fold in transgenic lines. Results also indicated that stress tolerance was improved due to a high frequency of autophagy and restriction of oxidative damage [263]. Overexpression of *Chrysanthemum* TF gene, *DgNAC1* increased salt tolerance in transgenic plants showing lower accumulation of MDA, H_2_O_2_, and O_2_^•−^, and significantly enhanced the activities of SOD, CAT, and POD [264]. Likewise, *PaSOD* from *Potentilla atrosanguinea* and *RaAPX* from *Rheum australe* were overexpressed in potato. Transgenic plants demonstrated enhanced activities of SOD and APX in dual transgenic plants (DTP). Superoxide dismutase and APX genes may serve as a positive regulator to increased salt tolerance by the regulation of ROS and lignin biosynthesis signaling pathways in transgenic plants [265]. Transgenic tobacco exhibited tolerance to heavy metals and overexpression of *SbMYB15* enhanced the activities of CAT and SOD, also increasing the expression of *MnSOD* at 100 μM (2-fold) and 300 μM (3-fold) of CdCl_2_, as well as *CAT1* by 62- and 9-fold at 100 and 300 μM of CdCl_2_ [266]. In regard to heavy metal tolerance, *CaGrx* from chickpea was overexpressed in *A. thaliana*. Transgenic plants showed maximal activities of GRX, GR, GPX, GST, and APX under AsIII and Cr stress compared with controls, whereas CAT, SOD, and MDHAR activities were also significantly increased. Authors suggested that *CaGrx* can be a suitable candidate gene to overcome metal stresses in other crops [267]. Overexpression of the *A. thaliana AtDREB1A* gene in tomato increased chilling tolerance. Transgenic plants enhanced the activities of SOD by 29% and CAT by 21%, indicating superior chilling stress tolerance [268]. As another example, overexpression of the potato *StSOD1* gene enhanced the activities of SOD, POD, and CAT under cold stress and improved cold tolerance in transgenic plants [269]. Similarly, the overexpression of *Chrysanthemum CmSOS1* gene enhances SOD and CAT by 171% in transgenic plants under WL conditions [270].

Interestingly, overexpression of *DaAPX* combined with supplementation of transgenic plants with H_2_O_2_ significantly enhanced the activity of APX, improving flooding and cold tolerance [217]. Therefore, a number of genes have been documented to provide significant protection against abiotic stress through genetic modification in several plant species; however, many of the genes are yet to be reported in major crops.

## 11. Conclusions and Perspectives

It is understood that abiotic stresses are major limiting factors affecting plant growth and development, globally. Thus, there is a growing interest in deciphering the physiological, biochemical, molecular, and cellular mechanisms of abiotic stress responses and tolerance and to introduce potential mitigation techniques that would enhance sustainable agricultural production. Abiotic stresses lead to the accumulation of ROS, which can be a source of oxidative injury in plants. Initially, ROS were considered as toxic molecules and products of aerobic metabolism, found in several subcellular compartments. The metabolism of ROS is crucial in crop growth, development, adaptation, and existence under stressful environments. The production and scavenging of ROS are essential factors of plant defense processes, and modulation and overexpression of candidate genes encoding ROS detoxifying enzymes are widely used to enhance tolerance against several abiotic stresses. However, the balance among the detoxification and generation of ROS is maintained by both enzymatic and nonenzymatic antioxidant systems under stressful conditions. Notably, ROS are known to perform a dual role in plant biology due to molecular cross-talk with other signaling molecules such as RNS, RSS, and RCS. Based on the literature, ROS is very important for various biological mechanisms, such as cellular proliferation and differentiation, and are known to exert a signaling role at low concentrations. However, ROS toxicity openly kills cells through oxidative stress, which is the outcome of ROS-activated pathways responsible for cell death.

In addition, interrelationship exists between ROS, RNS, RSS, and RCS metabolisms under normal and stressed situations; however, a few studies have been carried out to address these interactions. Both ROS and RNS can create oxidative and nitrosative stress solely or together nitro-oxidative stress; however, they are also involved in signaling process of higher plants especially under adverse environmental situations. On the other hand, both ROS and RSS signals are identical and signal through their reaction with Cys, however, RSS signaling appears to be more extensive than ROS signaling. Contrary, RCS can regulate ROS metabolism since these molecules are direct products of oxidative stress and have the potential to act as its sensors. Therefore, these four reactive molecules could be the new gateway of interests for the plant biologists. Although accumulation of knowledge related to signaling roles of these reactive molecules have been accelerated over the last decade, more detailed work is needed to elucidate their roles in plant stress responses.

With the recent progress in molecular and genetic tools, significant progress has been made in enhancing stress tolerance in plants through the development of transgenic plants with increased activities of antioxidant enzymes. Nevertheless, overexpression of genes encoding antioxidant enzymes in transgenic plants has a positive effect on abiotic stress tolerance and the increasing potential of antioxidant enzymes. Based on the available literature, there is a need to identify and report candidate genes that can considerably enhance the tolerance and yield of transgenic plants under stressful environments. In addition, chemical priming offers an attractive alternative to genetic engineering in order to achieve similar goals, often through the regulation of the antioxidant defense apparatus. In the future, systems biology approaches such as genomics, transcriptomics, proteomics, and metabolomics could aid us in introducing new ways for the development of stress tolerance. The integration of these approaches should be implemented to identify key and stress-related regulators, genes, proteins, and metabolites. Furthermore, identification and manipulation of pathways associated with ROS-detoxifying regulators can be improved to generate stress tolerance genotypes.

In the field environment, the plant has to face a variety of stresses at once; thus, identification of core genes, which can confer multiple abiotic stress tolerance, is of paramount importance. In addition, state-of-the-art genome-editing tools like CRISPR/Cas could help to modify the genome through the development of mutant plants with single or multiple genes (ROS-detoxifying regulators) for proper plant growth and development and to enhance the activities of antioxidant defense systems. Recently, speed breeding has also emerged as a powerful tool to boost the growth and development of plants under desired conditions. Therefore, to save time, genome editing could be coupled with speed breeding to develop transgenic plants with induced antioxidant apparatus that are stress tolerant and will thus contribute to feed millions and to ensure world food security.

## Figures and Tables

**Table 1:** Oxidative stress in plants under different abiotic stress factors.

Plant Species/Genotypes	Stress Condition	Oxidative Stress Status	References
**Salinity**			
*Triticum aestivum*	150 mM NaCl; 20 d applied on alternate days	62.11% and 63.78% increase in H_2_O_2_ and O_2_^•−^, respectively. 44% increase in lipid peroxidation.	[74]
*Vicia faba* cv. ILB-4347 and Hassawi-3	150 mM NaCl	90%, 66% and 84% increase in H_2_O_2_, MDA, and EL, respectively in ILB-4347. 128%, 92%, and 96% increase in H_2_O_2_, MDA, and EL, respectively, in Hassawi-3.	[124]
*Oryza sativa*	150 mM NaCl; 72 h	2-fold higher ROS level in root tissues. Sharp increases in lipid peroxidation, EL, H_2_O_2_, and O_2_^•−^ in leaf tissue.	[71]
*Solanum lycopersicum*	100 mM NaCl	157%, 176%, 158%, and 94% increased O_2_^•−^, H_2_O_2_, EL and MDA content.	[72]
*Vigna radiate*	100 mM NaCl	A 2-fold increase in the levels of H_2_O_2_, MDA, EL, and O_2_^•−^.	[74]
*Ocimum basilicum*	50 mM NaCl; sprinkling in 2 d intervals till harvest	Significant increase in H_2_O_2_ and MDA contents.	[21]
*Lens culinaris*	100 mM NaCl; 3 d	Higher accumulation of MDA and H_2_O_2_ content by 139% and 37%, respectively.	[125]
*Sorghum bicolor*	100 mM NaCl, 3 d intervals for 16 d	H_2_O_2_ content increased by 149% in leaf while 38% in roots. MDA content increased by 106% in leaf while 116% for roots.	[126]
*Chenopodium quinoa* cv. Q1, Q2, Q3, and Q4	100, 300, and 500 mM NaCl; 14 d	MDA and H_2_O_2_ increased in a concentration-dependent manner in all cultivars. Under 100 mM NaCl, the salt-sensitive cultivars (Q1, Q2, and Q3) showed the highest accumulation of H_2_O_2_ and MDA. Under 300 and 500 mM NaCl, cv. Q4 exhibited the least increase in MDA.	[127]
*Panicum italicum*	1% NaCl	1.5-fold increase in H_2_O_2_ and a 3-fold increase in MDA levels.	[128]
**Water Deficit and Simulated Drought**			
*Zea mays* cv. Run Nong 35, Wan Dan 13 and Dong Dan 0	Drought (80%, 60%, and 40% FC)	Increased ROS accumulation and membrane damage. Enhanced level of O_2_^•−^, H_2_O_2_, EL, LOX, and TBARS were found in all cultivars.	[129]
*Medicago sativa*	Water deficit, 7 d	Dramatically increased (by 5-fold) H_2_O_2_ content. Increased NO content (by 15%) compared with control.	[130]
*Brassica napus* Binasarisha-3	Osmotic stress (10% and 20% PEG), 48 h	Both levels of H_2_O_2_ and MDA were upregulated significantly, with the highest value in 20% PEG.	[89]
*B. napus* cv. Bulbul-98	Water deficit (30% FC)	Increased EL by 2-folds with membrane damage. Significant increase in H_2_O_2_ content.	[131]
*S. lycopersicum* mill. cv. Pusa 120	Drought (withheld irrigation), 6 d	Increased lipid peroxidation (MDA content) and EL (39%).	[88]
*V. radiata* cv. BARI Mung-2	Osmotic stress (5% PEG), 48 h	74% and 84% increase in H_2_O_2_ and O_2_^•^^−^ compared to control. 62% increase in LOX activity.	[91]
*S. bicolor* cv. Sugargraze	Water deficit, 16 d	113% increase in H_2_O_2_ content. Increased MDA content by 94% and 98% in leaf and root, respectively. A drastic loss in cell viability.	[126]
*T. aestivum*	Drought (35% FC)	31%, 25%, and 38% increase in TBARS, EL, and H_2_O_2_ contents, respectively, compared to control.	[92]
*B. napus* cv. BINA Sharisha-3	Osmotic stress (10% and 20% PEG), 48 h	123% and 93% increased MDA and H_2_O_2_ content over control.	[90]
*O. sativa,* sub1A quantitative trait loci (sub1A QTL)	Drought (withdrawing irrigation), 8 d	Increased O_2_^•−^, H_2_O_2_ and MDA content by 1.8-, 2.1-, and 1.66-folds, respectively.	[86]
*Eleusine coracana* L. Gaertn.	Drought (75% water deficit condition), 3 w	Increased EL and H_2_O_2_ content.	[87]
*B. rapa* cv. BARI Sharisha-15	Osmotic stress (20% PEG), 2 d	82% and 131% increased MDA and H_2_O_2_ content over control. Overproduction of toxic O_2_^•−^.	[132]
*Coffea arabica* L.	Drought (40% water holding capacity), 20 d	Increased MDA content.	[85]
*O. sativa* var. *japonica*. cv. Nipponbare	Osmotic stress (20% PEG-6000), 5 d	16% and 23% increased MDA and O_2_^•−^ accumulation comparing control. 1-fold enhanced H_2_O_2_ generation over control.	[93]
*Phragmites karka*	Drought (40% water holding capacity), 35 d	Increased MDA content by 22%.	[84]
*S. lycopersicum* cv. Login 935	Water deficit (60% FC), 20 d	83%, 37%, and 75% increased MDA, H_2_O_2,_ and O_2_^•^^−^ content compared to control.	[95]
*Glycine max*	Osmotic stress (15% PEG), 3 w	47% declined EL, while LOX activity enhanced by 38%.	[94]
**Toxic Metals/Metalloids**			
*O. sativa* cv. BRRI dhan54	0.25 and 0.5 mM NiSO_4_.7H_2_O, 3 d	Increased contents of MDA increased (by 172% and 199%). H_2_O_2_ (by 28% and 35%) and LOX activity (by 38% and 73%) under 0.25 mM and 0.5 mM Ni-stress, respectively.	[98]
*B. juncea* cv. BARI Sharisha-11	0.5 and 1.0 mM CdCl_2_, 3 d	Enhanced MDA content by 35% and 66%, H_2_O_2_ content by 43% and 54%, and LOX activity by 69% and 108% under 0.5 and 1.0 mM Cd stress, respectively.	[102]
*B. napus* BINA Sharisha-3	0.5 and 1.0 mM CdCl_2_, 2 d	Increased MDA contents by 56% and 133%, and H_2_O_2_ contents by 38% and 70% in 0.5 and 1.0 mM Cd stress, respectively.	[101]
*V. radiata* cv. BARI Mung-2	1.0 and 1.5 mM CdCl_2_, 2 d	Increased MDA level by 85% and 177%, H_2_O_2_ content by 73% and 127% and O_2_^•–^ generation rate by 69% and 120% under 1.0 and 1.5 mM Cd stresses, respectively.	[100]
*T. aestivum* cv. Pradip	0.5 and 1.0 mM Pb(NO_3_)_2_, 2 d	MDA content increased by 58% and 179% and H_2_O_2_ levels by 41% and 95% under both levels of Pb stress.	[107]
*V. radiata* cv. BARI mung-2	0.5 mM AlCl_3_, 2 and 3 d	H_2_O_2_, O_2_^•−^ generation rate, and LOX activity increased by 83%, 110%, and 72%, which increased the lipid peroxidation by 97%.	[133]
*B. juncea* cv. BARI Sharisha-11	0.15 and 0.3 mM K_2_CrO_4_, 5 d	Increased TBARS content (by 30% and 65%), H_2_O_2_ (by 24% and 46%), and LOX activity (by 68% and 101%) under both levels of Cr stress, respectively.	[104]
**Extreme Temperature**			
*Cucumis sativus*	35 ± 1 °C, 7 days	Increased MDA content (by 60.6%) and O_2_^−^ (by 79.9%).	[113]
*S. bicolor*	36/26 °C and 39/29 °C, until 7 d after full anthesis	O_2_^•−^ content increased 2 to 4-fold in pollen and 1 to 2.3-folds in the pistil.	[114]
*Gossypium hirsutum*	45/30 ± 2 °C, 120 d	Enhanced MDA content by 0.78%, affecting fiber quality and boll weight.	[134]
*O. sativa*	38 °C, 5 d	H_2_O_2_ accumulation increased 1-fold.	[93]
*O. sativa* cv. DM You 6188)	12 °C, for 6 d	Enhanced MDA content and EL by 180% and 49%, respectively.	[116]
*L. esculentum*	4 °C, 24 h	Enhanced H_2_O_2_ content by 32%.	[135]
*L. esculentum*. cv. C.H Falat	3 °C, 6 h, 6 d	Enhanced H_2_O_2_ content (by 2-fold) and EL (by 20%).	[136]
*Solanum lycopersicum* L.	15/8 °C day/night, 24 and 48 h	Increased MDA and H_2_O_2_ by 62% and 34%, respectively.	[117]
**Waterlogging**			
*S. bicolor* cvs. JN01 and JZ31	Waterlogged soil, 12 d	2.45-fold higher MDA content in WL-sensitive JZ31, but 1.8-fold higher in WL-tolerant JN01.	[120]
*Hordeum vulgare* cvs. TF57 and TF58	Waterlogged soil, 21 d	MDA content and O_2_^•–^ generation rate σ were markedly increased in WL-sensitive TF57, but slightly increased in WL-tolerant TF58 genotype.	[137]
*S. lycopersicum* cv. Roma	Waterlogged soil, 15 d	54% and 208% higher MDA and H_2_O_2_ contents, respectively.	[122]
*Deschampsia Antarctica*	Waterlogged soil, 7 d	84% and 52% higher MDA and H_2_O_2_ contents.	[123]
*Sesamum indicum* cv. BARI Til-4	Waterlogged soil, 2, 4, 6, and 8 d	Both MDA and H_2_O_2_ increased in a duration-dependent manner 39% and 62% higher MDA and H_2_O_2_ content after 8 days of WL.	[121]

**Table 2:** Reaction mechanisms of major reactive oxygen species (ROS) scavenging enzymatic antioxidants.

Antioxidants	Reactions Catalyzed	Catalytic Reaction Sites
	**Nonenzymatic**	
Ascorbic acid	Scavenges O_2_^•–^, H_2_O_2_, ^•^OH, and ^1^O_2_	Chloroplast, peroxisomes, cytosol, mitochondria, apoplast
Glutathione	Scavenges H_2_O_2_, ^•^OH, and ^1^O_2_	Chloroplast, peroxisomes, cytosol, mitochondria, apoplast
Tocopherol	Scavenges ^•^OH, ^1^O_2_, ROO^•^ and ROOH	Thylakoid membrane of chloroplast
Carotenoids	Scavenges mainly ^1^O_2_	Chloroplast
Flavonoids	Scavenges O_2_^•–^, H_2_O_2_, and ^1^O_2_	Chloroplast, vacuole
Phenolic acids	Scavenges O_2_^•−^, ^•^OH, ROO^•^, and ONOO^–^	Cell wall
Alkaloids	Scavenges O_2_^•–^, ^•^OH, H_2_O_2_, and ^1^O_2_	Vacuole
Nonprotein amino acids	Scavenges O_2_^•–^, H_2_O_2_, and ^1^O_2_	Cytosol, mitochondria, cell wall
	**Enzymatic**	
Superoxide dismutase (SOD; EC 1.15.1.1)	2O_2_^•−^ + 2H^+^→ O_2_ + H_2_O_2_	Chloroplast, peroxisomes, cytosol, mitochondria, apoplast
Catalase (CAT; EC 1.11.1.6)	2H_2_O_2_ → 2H_2_O + O_2_	Peroxisomes
Peroxidases (POX; EC 1.11.1.7)	2PhOH + H_2_O_2_→ 2PhO^•^ + 2H_2_O 2PhO^•^ → cross-linked substances PhO^•^ + Asc → PhOH + MDHA PhO^•^ + MDHA → PhOH + DHA	Cell wall, apoplast, vacuole
Polyphenol oxidase (PPO; EC 1.14.18.1)	PhOH + O_2_ → Catechols Catechols + O_2_ → Q + H_2_O	Thylakoid membrane of chloroplast, cytosol, vacuole
Ascorbate peroxidase (APX; EC 1.11.1.11)	H_2_O_2_ + AsA → 2H_2_O + MDHA	Chloroplast, peroxisomes, cytosol, mitochondria, apoplast
Monodehydroascorbate reductase (MDHAR; EC 1.6.5.4)	MDHA + NAD(P)H → AsA + NAD(P)^+^	Chloroplast, cytosol, mitochondria
Dehydroascorbate reductase (DHAR; EC 1.8.5.1)	2GSH + DHA → GSSG + AsA	Chloroplast, cytosol, mitochondria
Glutathione reductase (GR; EC 1.6.4.2)	GSSG + NADPH + H^+^ → GSH + NADP^+^	Chloroplast, cytosol, mitochondria
Glutathione peroxidase (GPX; EC 1.11.1.9)	H_2_O_2_ + GSH → H_2_O + GSSG	Cytosol, mitochondria
Glutathione *S*-transferase (GST; EC 2.5.1.18)	R-X + GSH → GS-R + H-X	Chloroplast, cytosol, mitochondria
Peroxiredoxins (PRX; EC 1.11.1.15)	H_2_O_2_ + PRX-S^–^ → OH^–^ + PRX-SOH PRX-SOH + GSH → PRX-SSG + H_2_O PRX-SSG + GSH → PRX-S^–^ + GSSG	Cytosol, chloroplasts, mitochondria, nucleus, extracellular spaces
Thioredoxin (TRX; EC 1.8. 1.9)	TRX-RS_2_ + NADPH + H^+^→ TRX-R(SH)_2_ + NADP^+^	Chloroplast, cytosol, mitochondria

**Table 3:** Antioxidant defense in plants under different abiotic stress factors.

Plant Species	Stress Conditions	Antioxidant Defense	References
**Salinity**			
*Triticum aestivum*	100 mM NaCl; 20 d	Nitrogen supplementations increased the activity of SOD, CAT, GR, MDHAR, and DHAR by 2-folds and APX 3-folds, respectively, compared to untreated.	[182]
*Nicotiana benthamiana*	150 mM NaCl; 15 d	Acetylcholine application increased SOD by 1-fold and POD by 2-folds.	[208]
*Solanum lycopersicum*	150 mM NaCl; 5 d	Vanillic acid increased AsA/DHA, GSH/GSSG, MDHAR, GR, GST, SOD, and CAT by 161%, 90%, 18%, 53%, 87%, 43%, 105%, respectively.	[209]
*Medicago sativa*	250 mM NaCl; 2 w	Melatonin increased the activities of CAT, POX, and Cu/Zn-SOD.	[210]
*Cucumis sativus*	150 mM NaCl; 3 d	Melatonin increased CAT, SOD, POD, and APX by 23%, 29%, 15%, and 16%, respectively.	[211]
*T. aestivum*	100 mM NaCl; 20 d	Sodium nitroprusside (SNP) and glucose solely increased Cys and GSH content by 86% and 79%, and 19% and 18%, respectively, whereas SOD, CAT, APX, and GR increased by 75% and 65%, 49% and 37%, 97% and 57%, and 60% and 57%, respectively. Combined SNP and glucose application increased the activity of these antioxidant enzymes (SOD, CAT, APX, and GR by 138%, 61%, 271%, 127% and 44%, 17%, 119%, 23%, respectively, compared to the control and glucose-treated plants.	[212]
*C. sativus*	200 mM NaCl; 7 d	H_2_S increased ASA content by 42.6% and GR activity by 9.1%. Reversed decreased SOD and POD activity.	[213]
*Brassica juncea*	100 mM NaCl; 15 d	Nitric oxide increased SOD, CAT, APX, and GR activity by 91%, 33%, 114%, and 49%, respectively.	[214]
**Water Deficit and Simulated Drought**			
*Zea mays* cv. Run Nong 35, Wan Dan 13 and Dong Dan 80	Mild drought (80% FC), moderate drought (60% FC), and severe drought (40% FC)	Increased activities of APX, MDHAR, and DHAR by 24%, 13%, and 29% in Dong Dan. 80% and 16%, 11%, and 10% in Wan Dan 13, respectively, under severe drought. Higher SOD activity as well as AsA and DHA contents under moderate and severe drought in both maize hybrids.	[129]
*Glycine max* and *G. tomentella*	Water deficit, flowering stage, 12 d	A substantially increased SOD and GR enzymes activities with the highest value during 8th day of stress treatment in *G. max*. A gradual increase in GR activity till the end of drought treatment was observed in *G. tomentella.*	[215]
*Brassica napus* cv. Binasarisha-3	Osmotic stress (10% and 20% PEG), 48 h	MDHAR activity was higher under 10% PEG. DHAR activity increased under both stress level. GR and GST activity was higher by 26% and 23% and 25% and 31% at both stress level, respectively.	[89]
*Phaseolus vulgaris* cv. Bn-150 (drought-tolerant) and Bn-16 (drought-sensitive)	Moderate drought (50% FC) and severe drought (0% FC), 14 d	Significantly increased total phenolic contents of Bn-150 by 223% and 265%, respectively, under moderate and severe drought. SOD, CAT, APX, and GR activities were increased in tolerant genotypes (Bn-150) than the sensitive one (Bn-16).	[97]
*Vigna radiata* cv. BARI Mung-2	Osmotic stress (5% PEG), 48 h.	Decreased AsA/DHA ratio by 54%. Increased APX and GR by 20% and 42%, respectively. Reduced CAT and MDHAR activity by 13% and 26%, respectively.	[91]
*B. napus* cv. Binasarisha-3	Osmotic stress (10% and 20% PEG), 48 h	Moderate stress increased AsA content, GPX, and GST activity but reduced CAT activity, whereas severe stress enhanced APX activity but reduced MDHAR, DHAR, and GR activities. Both levels of stress increased GSH and GSSG contents by 31% and 26%; and 83% and 225%, respectively, compared to control.	[90]
*B. rapa* cv. BARI Sharisha-15	Osmotic stress (20% PEG), 2 d	AsA and GSH contents increased by 10% and 72%, respectively. APX, GR, CAT, and GPX activity increased by 23%, 81%, 29%, and 26%, respectively.	[132]
*Z. mays* cv. Xida 889 and Xida 319	Drought (50% FC), 15 d	SOD and total antioxidant activities increased, whereas CAT, APX, and POD activity declined. GSH content increased by 17% and 28% in Xida 319 and Xida 889, respectively, compared to control.	[150]
*Oryza sativa* var. *japonica* cv. Nipponbare	Osmotic stress (20% PEG-6000), 5 d	Decreased SOD, APX, and CAT activities, but POD activity increased by 59% compared to control.	[93]
*S. lycopersicum* cv. Login 935	Drought stress (60% FC), 20 d	Enhanced SOD, CAT, and APX activities by 110%, 66%, and 77%, respectively. Increased AsA, GSH, and α tocopherol contents by 81%, 93%, and 103%, respectively.	[95]
*G. max*	Osmotic stress (5%, 10%, and 15% PEG), 3 w	Highest activities of CAT, APX, and PPO were observed at mild osmotic stress (5% PEG), whereas increased SOD and POX activities were found at severe osmotic stress (15% PEG). Total phenol and tocopherol contents increased by 51%, 32%, and 44%, and 26%, 26%, and 21% at three levels of osmotic stress intensities, respectively, compared to control.	[94]
**Toxic Metals/Metalloids**			
*Brassica napus* Cv. BINA Sharisha-3	0.5 and 1.0 mM CdCl_2_, 2 d	Reduction of AsA content, whereas higher GSSG content and GST activity. APX and GR activity increased, but CAT, MDHAR, and DHAR activity reduced.	[101]
*V. radiata* cv. BARI mung-2	0.5 mM AlCl_3_, 2 and 3 d	AsA content reduced, but GSH and GSSG increased. The activity of SOD, GST, GPX, APX, and GR increased, but MDHAR, DHAR, and CAT decreased.	[133]
*Oryza sativa* cv. BRRI dhan54	0.25 and 0.5 mM NiSO_4_⋅7H_2_O, 3 d	64% lower AsA and 146% higher GSH content at 0.5 mM Ni stress. APX, MDHAR, DHAR, and GR activities increased by 114%, 116%, 31%, and 104% at 0.5 mM Ni stress, respectively.	[98]
*Pisum sativum*	100 µM NiCl_2_, 3 d	GSH accumulation increased by 5-fold. SOD activity increased by 14-fold, CAT and APX activities both by 6-fold, and GR activity by almost 3-fold.	[99]
*P. sativum*	100 µM CdCl_2_, 3 d	GSH accumulation increased by 3-fold and GSSG by 2-fold. SOD activity increased by 10-fold, CAT and APX activities both by 8-fold, and GR activity by almost 4-fold.	[99]
*B. juncea* cv. BARI Sharisha-11	0.5 and 1.0 mM CdCl_2_, 3 d	42% lower AsA and 200% higher DHA at severe stress, whereas 44% and 72% higher GSSG content under mild and severe stress, respectively. 44% higher SOD and 31% higher GPX activity at severe stress.	[102]
*T. aestivum* cv. Pradip	0.5 and 1.0 mM Pb(NO_3_)_2_, 2 d	APX activity increased, but MDHAR and DHAR decreased; GR increased initially and then declined. 35% higher SOD, 44% higher GST along with 31% lower CAT, and 28% lower GPX activities were reported.	[107]
**Extreme Temperature**			
*Cicer arietinum* (sensitive genotype: ICC14183, ICC5912; tolerant genotypes: ICCV07110, ICCV92944)	30/20, 35/25, 40/30, and 45/35 °C; 2 d for flower and 8 d for three leaves stage	Reduced APX (by 38–49% and 43–50% at 40/30 °C) and GR (by 30–46% and 44–49% at 45/35 °C) activity as well as AsA (by 13–18% and 28–32% at 40/30 °C), and GSH (by 24–33% and 37–44% at 45/35 °C) content in sensitive genotypes.	[149]
*Cucumis sativus*	35 ± 1 °C; 7 d	Improved SOD (by 16.6%), CAT (by 13%), APX (by 25.2%), GR (by 14.4%), and POD (by 35.4%) activity.	[113]
*Sorghum bicolor*	36/26 and 39/29 °C; until 7 d after full anthesis	In pollen, decreased the SOD (58–87%), CAT (44–56%), and POX (36–60%) activity. In pistil, decreased the SOD (59–77%), CAT (35–60%), and POX (42–78%) activity.	[114]
*Gossypium hirsutum*	45/30 ± 2 °C; 120 d	Enhanced SOD and CAT activity.	[134]
*O. sativa*	38 °C; 5 d	Decreased the activity of SOD and CAT. Enhanced POD (by 32.1%) activity.	[93]
*O. sativa* cv. DM You 6188	12 °C; 6 d	Enhanced SOD (by 1.4%), CAT (by 1.58%), and GSH/GSSG (by 2.42-fold).	[116]
*Calendula officinalis*	4 °C; 24, 48, 72, 96, and 120 h	Elevated GR (161%), SOD (46%), and APX (82%) activity at 120 h.	[216]
*Capsella bursa-pastoris*	10 °C; 24, 48, 72, 96, and 120 h	Elevated GR (70%), POD (79%), and CAT (70%) activity at 120 h.	[202]
*Citrus reticulata*	1, −1, and −3 °C; 3 h	Enhanced CAT (1.35-fold) and APX (2-fold) activities.	[203]
*Vitis vinifera*	5 °C; 6, 12, 24, 48, and 72 h	Elevated GR (20.26%), DHAR (7.64%), and MDHAR (16.60%) activities with increased AsA (12.13%), DHA (7.89%), and GSH (56.09%) contents.	[217]
**Waterlogging**			
*S. bicolor* cv. JN01 and JZ31	Waterlogged soil, 12 d	Increased SOD (by 1.38- and 1.5-fold) and CAT (by 1.43- and 1.36-fold) in JN01 and JZ31, respectively.	[120]
*Hordeum vulgare* cvs. TF57 and TF58	Waterlogged soil, 21 d	SOD, POD, and CAT activities increased in both WL-sensitive TF57 and WL-tolerant TF58 genotype.	[137]
*S. lycopersicum* cv. Roma	Waterlogged soil, 15 d	AsA content reduced by 31%. SOD, CAT, and POD activities increased by 7%, 33%, and 57%, respectively, compared with control samples.	[122]
*Deschampsia antarctica*	Waterlogged soil, 7 d	Increment of CAT activity by 91%.	[123]
*Sesamum indicum* cv. BARI Til-4	Waterlogged soil, 2, 4, 6, and 8 d	GSH and GSSG increased by 45% and 150%, respectively, whereas AsA content decreased by 38% after 8 d WL. APX and MDHAR activity increased by 61% and 55%, but DHAR and GR activity reduced by 59% and 23%, respectively, after 8 d WL.	[121]

**Table 4:** Effect of exogenous H_2_O_2_ in plants under different abiotic stress factors.

Plant Species	Stress Condition	H_2_O_2_ Treatments	Positive Effects	References
*Triticum aestivum* cv. Zhengmai No. 004	150 mM NaCl; 2 d	Cotreatment; 0.05 µM, 2 d	Decreased MDA content and O_2_^•−^ generation. Increased GSH and carotene content by 21% and 33%, respectively. Increased SOD, POD, CAT, and APX activity. Increased growth and biomass.	[229]
*Cucumis sativus* cv. Jinchun no. 4 and Lvfeng no. 6	Osmotic stress; (10% PEG 6000); 2 d	Pretreatment as spraying; 1.5 mM	Decreased MDA and H_2_O_2_ content. Increased AsA and GSH content. Increased activity of GPX, CAT, APX, GR, MDHAR, and DHAR.	[230]
*C. sativus* cv. Jinchun no. 4	Low light; 100 mol m^−2^ s^−1^; 144 h	Pretreatment as spraying; 1.5 mM	Decreased O_2_^•−^, H_2_O_2_, and MDA content. Increased CAT, SOD, APX, GR, MDHAR, and DHAR activity.	[231]
*Vigna radiata* L. Wilczek) cv. SML-668	Cu, (CuSO_4_·5H_2_O); 50 and 100 mg kg^−1^ of soil.	Spraying; 2.5 mM	Increased relative water content (RWC) and SPAD value. Increased Pro content. Enhanced activity of SOD and CAT. Increased growth.	[16]
*Zea mays*	Osmotic stress (3% PEG 6000), 12 h	Pretreatment; 10 mM, 6 h	Decreased water loss, MDA, and H_2_O_2_ content. Increased levels of soluble sugars and proline. Increased Put, Spd, and Spm content by 72%, 106%, and 68%, respectively, over control.	[15]
*Glycine max* cv. Merrill 537	Drought; withholding irrigation, 4 and 7 d	Foliar spray; 1 mM, 3 d	Improved water status, pigment content, and alleviated lipid peroxidation. Decreased MDA and H_2_O_2_ content. Increased activity of SOD (by 93% and 190%), CAT (by 49% and 120%), APX (by 106% and 194%), and GR (by 31% and 229%) on day 4 and 7 of the drought period, respectively, over control.	[17]
*C. sativus* L.	Drought; 60 ± 5% FC	Spraying; 1.5 mM (100 mL pot^−1^)	Decreased MDA content and ROS (O_2_^•−^, H_2_O_2_) generation. Increased activity of SOD and POD. Increased soluble sugar and proline content. Increased chl and RWC.	[18]
*Brassica napus* cv. Binasarisha-3	Cd; 0.5 mM (mild) and 1.0 mM (severe) CdCl_2_; 2 d	Pretreatment; 50 µM, 24 h	Decreased MDA content (by 23% and 25%) under both Cd toxicity levels, respectively. Decreased H_2_O_2_ content. Increased AsA (by 32% and 30%); GSH content (by 38% and 25%) under both Cd stresses, respectively. Enhanced GSH/GSSG ratio. Increased activity of APX (by 40% and 39%), DHAR (by 77% and 67%), GR (by 36% and 79%), GST (by 44% and 43%), CAT (by 79% and 47%), and glyoxalase II (by 47% and 55%) under both Cd stresses, respectively. Enhanced GPX activity (by 40%) under severe stress and glyoxalase I activity (by 35%) under mild stress.	[22]
*T. aestivum* cv. Fsd-2008 and S-24	Drought; withholding irrigation, 6 w	Seed priming; 1.5 mM, 16 h	Decreased MDA and H_2_O_2_ content. Enhanced the activity of SOD, CAT, and POD. Increased photosynthetic pigments. Increased GB and Pro content.	[14]
*Oryza sativa* cv. BRRI dhan29	Osmotic stress (15% PEG-6000)	Foliar spray; 5 and 10 mM	Decreased MDA and H_2_O_2_ content. Increased activity of CAT and GPX. Protected photosynthetic pigments.	[13]

**Table 5:** Recent advancements in transgenic approaches to enhance the activities of antioxidant defense systems under abiotic stress conditions. Described studies increased stress tolerance by reducing the damage of oxidative stress and by increasing scavenging of ROS under stressful conditions.

Stress Condition and Duration	Studied Plant	Source Plant	Gene	Impact on Antioxidant Defense Systems	References
**Salinity**					
0, 50, 100, and 150 mM NaCl; 7 and 15 d	*Solanum tuberosum*	*Potentilla atrosanguinea* and *Rheum australe*	*PaSOD* and *RaAPX*	Enzyme activities are enhanced in transgenic plants as of SOD by 2–6-fold in *PaSOD* and 1–3-fold in double transgenic plants (*DTP*); APX by 5–11-fold in *APX* and 4–8 fold in *DTP.*	[265]
100, 200, and 400 mM; 1, 5, 10, 15 d	Chrysanthemum	*Dendronthema grandiform*	*DgNAC1*	Enzyme activities are enhanced in transgenic plants as of SOD by 2-, CAT by 2-, and POD by 3-folds.	[264]
150 mM NaCl; 3, 6, 9, 12, and 24 h	*Arabidopsis thaliana*	*Vitis vinifera*	*VvWRKY30*	Enzyme activities are significantly enhanced in transgenic plants, i.e., POD, CAT, and SOD.	[271]
150 or 200 mM NaCl, till germination	*Glycine max*	*Glycine max*	*GmMYB84*	Enzyme activities are significantly enhanced in transgenic plants, i.e., SOD, POD, and CAT.	[272]
**Water Deficit and Simulated Drought**					
Osmotic stress (20% PEG); 1, 3, 6 12, 24, and 48 h	*Nicotiana tabacum*	*Spinacia oleracea*	*SoCYP85A1*	Overexpressed lines improve the activity of POD by 1.3–1.5 and SOD by 1.36–1.39-fold	[273]
Withholding water for 14 and 21 d	*Malus domestica*	*Malus domestica*	*MdATG18a*	Enzyme activities are enhanced in transgenic plants as of CAT and POD by 1.57–2.05-fold in overexpressed lines.	[263]
Withholding water till the wilting stage	*Arachis hypogaea*	*Macrotyloma uniflorum* Lam. Verdc.	*MuWRKY3*	Enzyme activities are enhanced in transgenic plants as of SOD by 3–5 and APX by 3-7–fold	[262]
Osmotic stress (15% PEG); 60 d	*N. tabacum*	*Zea mays*	*ZmSO*	Overexpressed lines increase the activity of GSH 64% and 88%.	[274]
Osmotic stress (15% and 25% PEG); 7 d	*A. thaliana*	*Cicer arietinum*	*CaMT*	Enzyme activities are enhanced in transgenic plants as of APX 488%, POD 135%, GPX 134%, and GRX 186%.	[275]
Withholding water for 12 d	*A. thaliana*	*Malus prunifolia*	*MpDGK2*	Enzyme activities are enhanced in transgenic plants, i.e., CAT, APX, and POD.	[276]
**Toxic Metals/Metalloids**					
As(III) (5 and 10 μM (NaAsO_2_), As(V) (50 and 100 μM (Na_2_HAsO_4_), Cd (30 and 50 μM (CdCl_2_) and Cr (K_2_Cr_2_O7)	*A. thaliana*	*Oryza sativa*	*OsSultr1;1*	Enzyme activity is enhanced in transgenic plants, i.e., GSH with As(III) toxicity.	[277]
300 μM CdCl_2_ and 300 μM NiCl_2_.6H_2_O; 1, 12, 24, and 48 h	*N. tabacum*	*Salicornia brachiate*	*SbMYB15*	Enzyme activities are enhanced in transgenic plants as of CAT and SOD and also increase the expression of *MnSOD* at 100 μM (1.69-fold) and 300 μM (3.2-fold) of CdCl_2_ and *CAT1* by 62.19- and 9.8-fold at 100 and 300 μM.	[266]
**Extreme Temperature**					
45 °C; 0.5, 1, 2, 3, 6, 9, 12, and 24 h	*N. tabacum*	*Triticum aestivum*	*TaFBA1*	Enzyme activities are enhanced in transgenic plants, i.e., SOD, POD, and APX, while CAT activity was decreased under heat stress.	[278]
48 °C; 6 h	*M. domestica*	*M. domestica*	*MdATG18a*	Enzyme activities are enhanced in transgenic plants, i.e., SOD, POD, CAT, AsA, and GSH, whereas a decreased ratio of GSH/GSSG was reported.	[279]
4 °C; 5 d	*Solanum lycopersicum*	*A. thaliana*	*AtDREB1A*	Enzyme activities are enhanced in transgenic plants as of SOD by 29.49% and CAT by 21.34%.	[268]
4 °C; 6, 12, 24, 36, and 48 h	*S. tuberosum*	*S. tuberosum*	*StSOD1*	Enzyme activities are enhanced in transgenic plants as of SOD by 1.38, POD by 1.24, and CAT by 1.37 folds.	[269]
**Waterlogging**					
2 cm waterlogging; 3, 6, 12, 24, and 72 h	*A. thaliana*	*Brassica napus*	*BnERF2.4*	Enzyme activities are enhanced in transgenic plants, i.e., SOD, POD, and CAT.	[280]
Soil–atmosphere interface for 1 w	*A. thaliana*	*Mentha arvensis*	*MaRAP2-4*	Enzyme activities are enhanced in transgenic plants, i.e., CAT, GPX, and SOD.	[281]
5 cm waterlogging; 24 and 48 h	*A. thaliana*	*Dioscorea alata*	*DaAPX*	Enzyme activity is enhanced in transgenic plants, i.e., APX but no significant effect on CAT.	[217]
3 cm standing water; 14 d	*Chrysanthemum morifolium*	*Chrysanthemum morifolium*	*CmSOS1*	Overexpressed plants enhance the activities of SOD and CAT by 171%.	[270]
3.0 cm above the nutritional substance surface; 2 weeks	*A. thaliana*	*Hordeum vulgare*	*HvERF2.11*	Enzyme activities are enhanced in transgenic plants as of SOD by 55%, 48%, and 45%, POD by 64%, 65%, and 70%, CAT by 2.2%, 2.1%, and 2.1%, alcohol dehydrogenases by 2.1-, 2.3-, and 1.9-fold in three transgenic lines, respectively.	[282]

## Abbreviations

ABA—Abscisic acid; AEGs—Advanced glycation end products; AO—Aldehyde oxidase; RO^•^—Alkoxy radical; Al—Aluminum; As—Arsenic; APX—Ascorbate peroxidase; AsA—Ascorbic acid; AUX—Auxin; Cd—Cadmium; CAT—Catalase; Chl—Chlorophyll; Cr—Chromium; CA—Citric acid; Cu—Copper; Cys—Cysteine; DHA—Dehydroascorbate; DHAR—Dehydroascorbate reductase; DTP—Dual transgenic plants; EL—Electrolyte leakage; ETC—Electron transport chains; ET—Ethylene; RO*—Excited carbonyls; GRX—Glutaredoxin; GPX—Glutathione peroxidase; GR—Glutathione reductase; GST—Glutathione *S*-transferase; GOX—Glycolate oxidase; GOPX—Guaiacol peroxidase; HNE—4-Hydroxy-2(E)-nonenal; HT—High temperature; RH—Hydrocarbon; H_2_O_2_—Hydrogen peroxide; H_2_S—Hydrogen sulfide; ROOH—Hydroperoxides; HO_2_^•^—Hydroperoxyl radical; ^•^OH—Hydroxyl radical; HOBr—Hypobromous acid; HOCl—Hypochlorous acid; HOI—Hypoiodous acid; JA—Jasmonic acid; Pb—Lead; LOX—Lipoxygenases; LT—Low temperature; MDA—Malondialdehyde; MG—Methylglyoxal; MAPK—Mitogen-activated protein kinase; MDHA—Monodehydroascorbate; MDHAR—Monodehydroascorbate reductase; AOX—NADPH oxidase-like alternative oxidase; Ni—Nickel; NADPH—Nicotinamide adenine dinucleotide phosphate; NR—Nitrate reductase; NO—Nitric oxide; GSSG—Oxidized glutathione; OEC—Oxygen-evolving complex; O_3_—Ozone; POD/POX—Peroxidase; PRXs—Peroxiredoxins; ROO^•^—Peroxyl radical; ONOO^–^—Peroxynitrite; PhOH—Phenolic compounds; PhO^•^—Phenoxyl radical; pQ—Photochemical quenching; PS I—Photosystem I; PS II—Photosystem II; PSII-LHC—Photosystem II—light harvesting complex; PSII RC—Photosystem II—reaction center; PCs—Phytochelatins; PA—Polyamines; PEG—Polyethylene glycol; PPO—Polyphenol oxidase; PTM—Post-translational modification; PCD—Programmed cell death; Q_A_—Quinone; RCS—Reactive carbonyl species; RNS—Reactive nitrogen species; ROI—Reactive oxygen intermediates; ROS—Reactive oxygen species; RSS—Reactive sulfur species; GSH—Reduced glutathione; RWC—Relative water content; SA—Salicylic acid; SQ^•−^—Semiquinone; Si—Silicon; ^1^O_2_—Singlet oxygen; GSNO—*S*-nitrosoglutathione; NaCl—Sodium chloride; NaHS—Sodium hydrosulfide; –SOH—Sulfenic acid; O_2_^•−^—Superoxide; SOD—Superoxide dismutase; TBARS—Thiobarbituric acid reactive substances; –SH—Thiolate; TRXs—Thioredoxins; ^3^O_2_—Triplet oxygen; UO—Urate oxidase; WL—Waterlogging; WT—Wild-type; XOD—Xanthine oxidase.
